# A homozygous *KAT2B* variant modulates the clinical phenotype of *ADD3* deficiency in humans and flies

**DOI:** 10.1371/journal.pgen.1007386

**Published:** 2018-05-16

**Authors:** Sara Gonçalves, Julie Patat, Maria Clara Guida, Noelle Lachaussée, Christelle Arrondel, Martin Helmstädter, Olivia Boyer, Olivier Gribouval, Marie-Claire Gubler, Geraldine Mollet, Marlène Rio, Marina Charbit, Christine Bole-Feysot, Patrick Nitschke, Tobias B. Huber, Patricia G. Wheeler, Devon Haynes, Jane Juusola, Thierry Billette de Villemeur, Caroline Nava, Alexandra Afenjar, Boris Keren, Rolf Bodmer, Corinne Antignac, Matias Simons

**Affiliations:** 1 Laboratory of Hereditary Kidney Diseases, Institut National de la Santé et de la Recherche Médicale (Inserm) UMR1163, *Imagine* Institute, Paris, France; 2 Laboratory of Epithelial Biology and Disease, Institut National de la Santé et de la Recherche Médicale (Inserm) UMR1163, *Imagine* Institute, Paris, France; 3 Université Paris Descartes—Sorbonne Paris Cité, *Imagine* Institute, Paris, France; 4 Development, Aging and Regeneration Program, Sanford-Burnham-Prebys Medical Discovery Institute, La Jolla, CA, United States of America; 5 Department of Medicine IV, Medical Center–University of Freiburg, Faculty of Medicine, University of Freiburg, Freiburg, Germany; 6 Department of Pediatric Nephrology, Centre de Référence des Maladies Rénales Héréditaires de l'Enfant et de l'Adulte (MARHEA), Hôpital Necker-Enfants Malades, Assistance Publique—Hôpitaux de Paris (AP-HP), Paris, France; 7 Department of Genetics, Hôpital Necker-Enfants Malades, AP-HP, Paris, France; 8 BIOSS Center for Biological Signalling Studies and Center for Systems Biology (ZBSA), Albert-Ludwigs-University, Freiburg, Germany; 9 III. Department of Medicine, University Medical Center Hamburg-Eppendorf, Hamburg, Germany; 10 Division of Genetics, Arnold Palmer Hospital for Children, Orlando Health, Orlando, FL, United States of America; 11 GeneDx, Inc, Gaithersburg, MD, United States of America; 12 Sorbonne Université, UPMC, GRC ConCer-LD and AP-HP, Hôpital Trousseau, Service de Neuropédiatrie—Pathologie du développement, Paris, France; 13 Centre de référence des déficits intellectuels de causes rares, Inserm U 1141, Paris, France; 14 Sorbonne Universités, UPMC Univ Paris 06, Inserm U1127, CNRS UMR 7225, Institut du Cerveau et de la Moèlle Épinière (ICM), Paris, France; 15 AP-HP, GH Pitié-Salpêtrière, Department of Genetics, Unit of Developmental Genomics, Paris, France; 16 AP-HP, Hôpital Trousseau, Centre de référence des malformations et maladies congénitales du cervelet, Département de génétique et embryologie médicale, Paris, France; Baylor College of Medicine, UNITED STATES

## Abstract

Recent evidence suggests that the presence of more than one pathogenic mutation in a single patient is more common than previously anticipated. One of the challenges hereby is to dissect the contribution of each gene mutation, for which animal models such as *Drosophila* can provide a valuable aid. Here, we identified three families with mutations in *ADD3*, encoding for adducin-γ, with intellectual disability, microcephaly, cataracts and skeletal defects. In one of the families with additional cardiomyopathy and steroid-resistant nephrotic syndrome (SRNS), we found a homozygous variant in *KAT2B*, encoding the lysine acetyltransferase 2B, with impact on KAT2B protein levels in patient fibroblasts, suggesting that this second mutation might contribute to the increased disease spectrum. In order to define the contribution of *ADD3* and *KAT2B* mutations for the patient phenotype, we performed functional experiments in the *Drosophila* model. We found that both mutations were unable to fully rescue the viability of the respective null mutants of the *Drosophila* homologs, *hts* and *Gcn5*, suggesting that they are indeed pathogenic in flies. While the *KAT2B/Gcn5* mutation additionally showed a significantly reduced ability to rescue morphological and functional defects of cardiomyocytes and nephrocytes (podocyte-like cells), this was not the case for the *ADD3* mutant rescue. Yet, the simultaneous knockdown of *KAT2B* and *ADD3* synergistically impaired kidney and heart function in flies as well as the adhesion and migration capacity of cultured human podocytes, indicating that mutations in both genes may be required for the full clinical manifestation. Altogether, our studies describe the expansion of the phenotypic spectrum in *ADD3* deficiency associated with a homozygous likely pathogenic *KAT2B* variant and thereby identify *KAT2B* as a susceptibility gene for kidney and heart disease in *ADD3-*associated disorders.

## Author summary

Genetic diseases with complex syndromic constellations may be caused by mutations in more than one gene. Most examples studied so far describe genetic interactions of known disease genes, suggesting that a large number of multilocus diseases remain unexplored. Assessment of mutation pathogenicity can be achieved using animal models. One main advantage of using *Drosophila* is that it allows easy *in vivo* gene manipulation in cell types that are relevant for the disease. Here, we report the pathogenicity of *ADD3* mutations in three families with intellectual disability, microcephaly, cataracts and skeletal defects. Moreover, we provide evidence that the renal and cardiac phenotypes in one of the families could be unmasked by a homozygous variant in the lysine acetyltransferase encoding *KAT2B* gene. In *Drosophila*, this variant resulted not only in decreased viability, but also in functional defects in cardiomyocytes and nephrocytes, the latter being similar to mammalian podocytes. Our study implicates *KAT2B* as a susceptibility gene for steroid-resistant nephrotic syndrome (SRNS) and cardiomyopathy and emphasizes the importance of protein acetylation in kidney and heart function.

## Introduction

The interrogation of the entire genome via next generation sequencing (NGS) technology has revolutionized clinical diagnostics. For medical genetics that traditionally focuses on finding monogenetic causes for Mendelian diseases, NGS has not only introduced much higher mutation detection rates but also unprecedented complexities. A recent retrospective analysis of more than 7000 exomes revealed multiple molecular diagnoses in around five percent of cases with suspected monogenic disease [1], suggesting that patients with multilocus diseases are underrecognized.

The phenotypic complexity of multilocus diseases, of which digenic disease represents the simplest and most common form, can be challenging for the physician, both when it comes to finding a diagnosis and to genetic counseling and risk assessment. Two distinct disease phenotypes in a single patient may present with a completely new clinical phenotype. On the other hand, two overlapping disease phenotypes may be misinterpreted as a single disease with increased severity. The underlying genetic defects are equally difficult to predict. Both compound phenotypes caused by mutations in two completely unrelated genes [1] and overlapping disease phenotypes caused by mutations in genes within the same pathway are possible [2–4]. But as genes can be pleiotropic, there are most likely many exceptions to this. Also, while two loci may be equal in importance [2], a second variant may simply enhance the general or organ-specific penetrance of a given mutation [4].

One important challenge is therefore to decompose the contributions of each gene mutation or variant to the clinical phenotypes in question. So far, most reports on digenic inheritance in Mendelian disease have focused on known disease genes [1, 5, 6]. However, the diagnosis is even more difficult when dealing with genes that have previously not been associated with any genetic diseases.

In this study, we identify three families with mutations in *ADD3*, encoding for adducin-γ, with intellectual disability, microcephaly, cataracts and skeletal defects, further supporting that *ADD3* is a disease gene as previously reported for a single family [7]. We further use mutation validation in *Drosophila* and mammalian cell culture to demonstrate that in one of the families additional phenotypes in kidney and heart are associated with a homozygous missense variant in the lysine acetyltransferase *KAT2B*.

## Results

### Clinical features of three families with intellectual disability and microcephaly

**Fig 1:**
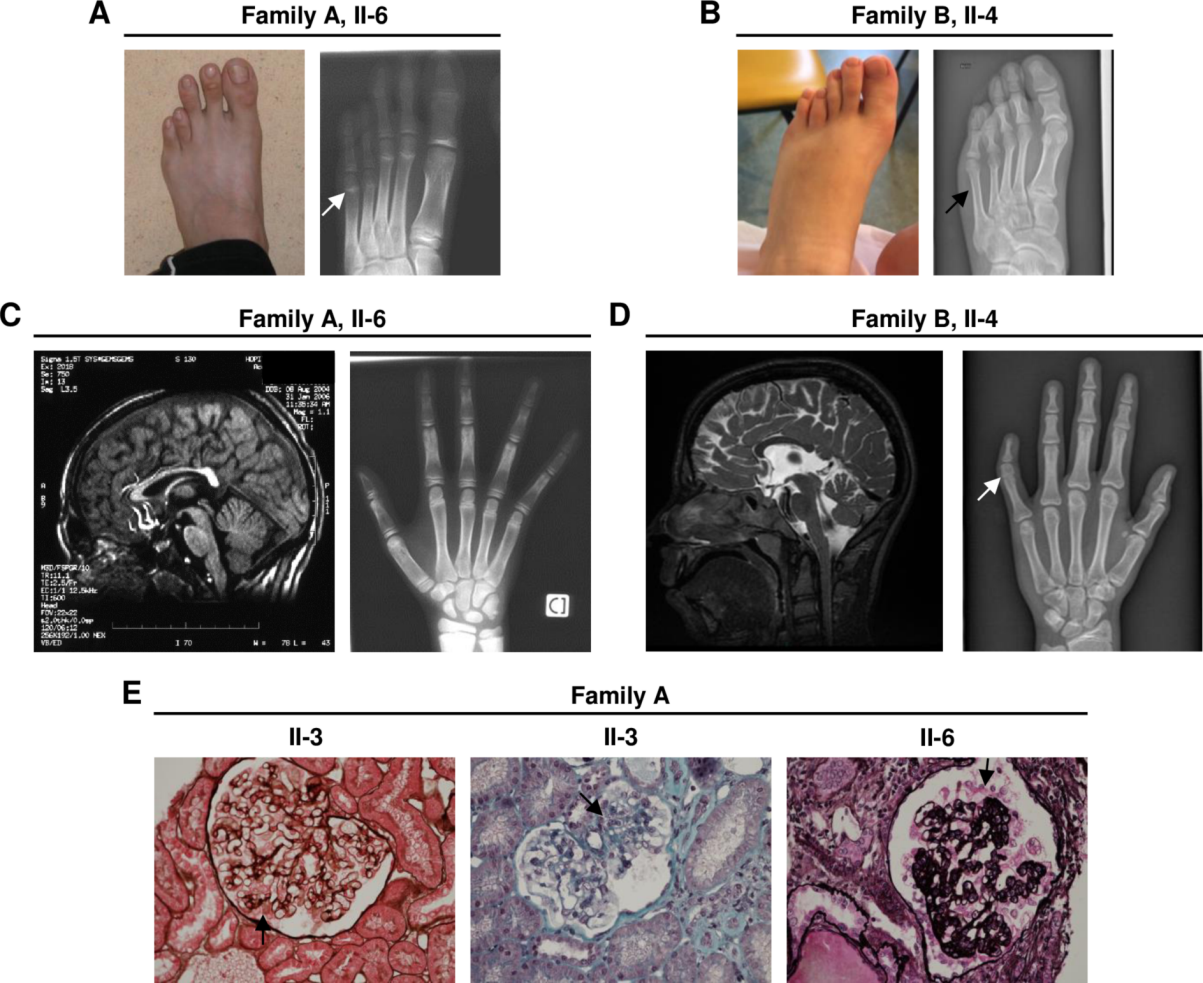
Clinical phenotypes of affected individuals. (A, B) Individuals II-6 (A; family A) and II-4 (B; family B) both show short 4^th^ and 5^th^ metatarsals. (C) Individual II-6 (family A) shows thin corpus callosum in the brain MRI at 17 months as well as hand arachnodactyly in the skeletal radiography. (D) Individual II-4 (family B) shows partial agenesis of the corpus callosum in the brain MRI at 14 years as well as mid phalanx hypoplasia in digit V (arrow) in the skeletal radiography. (E) Kidney sections of affected individuals from family A. At 15 years old, 3 years after the proteinuria onset, individual II-3 showed mostly normal glomeruli but with hypertrophic podocytes (arrow) (left panel; methenamine-silver stain; 40x), while few glomeruli (middle panel) had segmental sclerosis of the glomerular tuft (arrow; trichrome stain; 40x). There were no major lesions of the tubular-interstitial compartment (left and middle). Individual II-6 (right panel) presented with end-stage renal disease at 13 years old and showed severe glomerulosclerosis of almost all the glomeruli with retraction of the glomerular tuft and hypertrophic podocytes (arrow) (methenamine-silver stain; 40x).

Six individuals in three families (families A-C) with intellectual disability and varying degrees of microcephaly (Table 1) were identified for this study. Individuals from family A and B also shared bilateral cataracts, corpus callosum defects as well as specific skeletal defects such as shortening of the third and fourth metatarsals (Fig 1A–1D and Table 1), while the affected boy from family C suffered from epilepsy, severe speech delay and suspected cerebral palsy (Table 1).

**Table 1:** Clinical phenotype of affected individuals.

	Family A			Family B		Family C	Kruer et al (3)
	II-1	II-3	II-6	II-3	II-4	II-1	4 affected sibs (II-1, II-2, II-3, II-4)
**Sex**	F	F	M	NK	F	M	II-1 and II-3: F II-2 and II-5: M
**SRNS**	Yes	Yes	Yes	NK	No	No	No
**Age of onset of proteinuria (yrs)**	7	12	<13	NA	No proteinuria	No proteinuria	NA
**Renal histology**	FSGS	FSGS	FSGS	NA	NA	NA	NA
**Age of ESRD (yrs)**	17	27	13	NA	NA	NA	NA
**Heart disease**	Dilated cardiomyopathy (dx 16 yrs), supra-ventricular arrhythmia (frequent auricular extra-systoles), heart failure	Dilated cardiomyopathy, arrythmia	Dilated cardiomyopathy (dx 8 yrs), arrhythmia (ventricular hyperexcitation), heart failure	NK	No	No	No
**Neurological features**	*Borderline microcephaly*. Intellectual disability. MRI–aspects of global demyelination. *Axonal demyelinating motor*-sensory *neuropathy*	CP: -1SD. Intellectual disability.	*Borderline microcephaly (*CP: -2SD). Intellectual disability. MRI–thin corpus callosum.	Corpus callosum agenesis	Microcephaly (CP: -3SD), Intellectual disability–moderate. MRI–partial agenesis of corpus callosum.	Microcephaly (CP: -2.4 SD), Intellectual disability. Intractable seizures. MRI–possible cortical dysplasia	Borderline microcephaly (all sibs). Intellectual disability—mild to severe (all sibs). Spastic plegia (all sibs). Thin corpus callosum (II-2). Supranuclear gaze palsy (II-2). Epilepsy (II-2). Convergence-retraction nystagmus and strabismus (II-5). Strabismus (II-3)
**Cataract**	Congenital bilateral cataract	Congenital bilateral cataract	Bilateral cataract (6 yrs)	NK	Bilateral cataract	No	NK.
**Other features**	Mild facial dysmorphy (wide nasal bridge). *Arachnodactyly*, lax joints, cubitus valgus, scoliosis. Short stature.	Dysmorphic features similar to the two brothers	Facial dysmorphy (wide nasal bridge, slight proptosis). *Arachnodactyly*, short 4^th^ and 5^th^ metatarsals, conical phalanges. Lax joints, cubitus valgus scoliosis, spread iliac wings, short femural neck. Microcytic anemia	NK	Mild facial dysmorphy (wide nasal bride, bulbous nasal tip, narrow palpebral fissures). Fifth finger mid-phalanx hypoplasia, short 4^th^ and 5^th^ metatarsals. Short stature.	Facial dysmorphy. Short stature	
**Age at last examination *vs* † age (yrs)**	† 19	† 28	19.	TOP	14	8	16 (II-1) 13 (II-2) 9(II-3) 3(II-5)

Abbreviations are as follows: CP, cephalic perimeter; ESRD, end-stage renal disease; F, female; FSGS, focal segmental glomerulosclerosis; yrs, years; M, male; MRI, magnetic resonance imaging NA, not applicable; NK, not known; SRNS, steroidresistant nephrotic syndrome; SD, standard deviation; TOP, termination of pregnancy; yrs, years

†, deceased

The affected sibs in the consanguineous family A additionally presented with steroid-resistant nephrotic syndrome (SRNS), a progressive renal disease characterized by podocyte lesions and massive proteinuria [8], and cardiomyopathy (Table 1). For individual II-1 and II-3, proteinuria was first detected at 7 and 12 years of age, respectively, and end-stage renal disease was diagnosed a decade later. Individual II-6 was diagnosed with SRNS and end-stage renal disease at the age of 13 years. In kidney biopsies, individuals II-3 and II-6 (Fig 1E) both showed focal segmental glomerulosclerosis (FSGS). In the biopsy of individual II-6, whose renal disease was at a more advanced stage, hypertrophic and vacuolated podocytes (Fig 1E) as well as tubular atrophy, interstitial fibrosis and inflammatory cell infiltrates could also be found. In addition, all affected individuals from family A developed dilated cardiomyopathy with progressive heart failure and arrhythmia (Table 1). Cardiac failure was the cause of death for both individuals II-1 and II-3.

### Whole-exome sequencing identifies missense mutations in *ADD3* in all families

**Fig 2:**
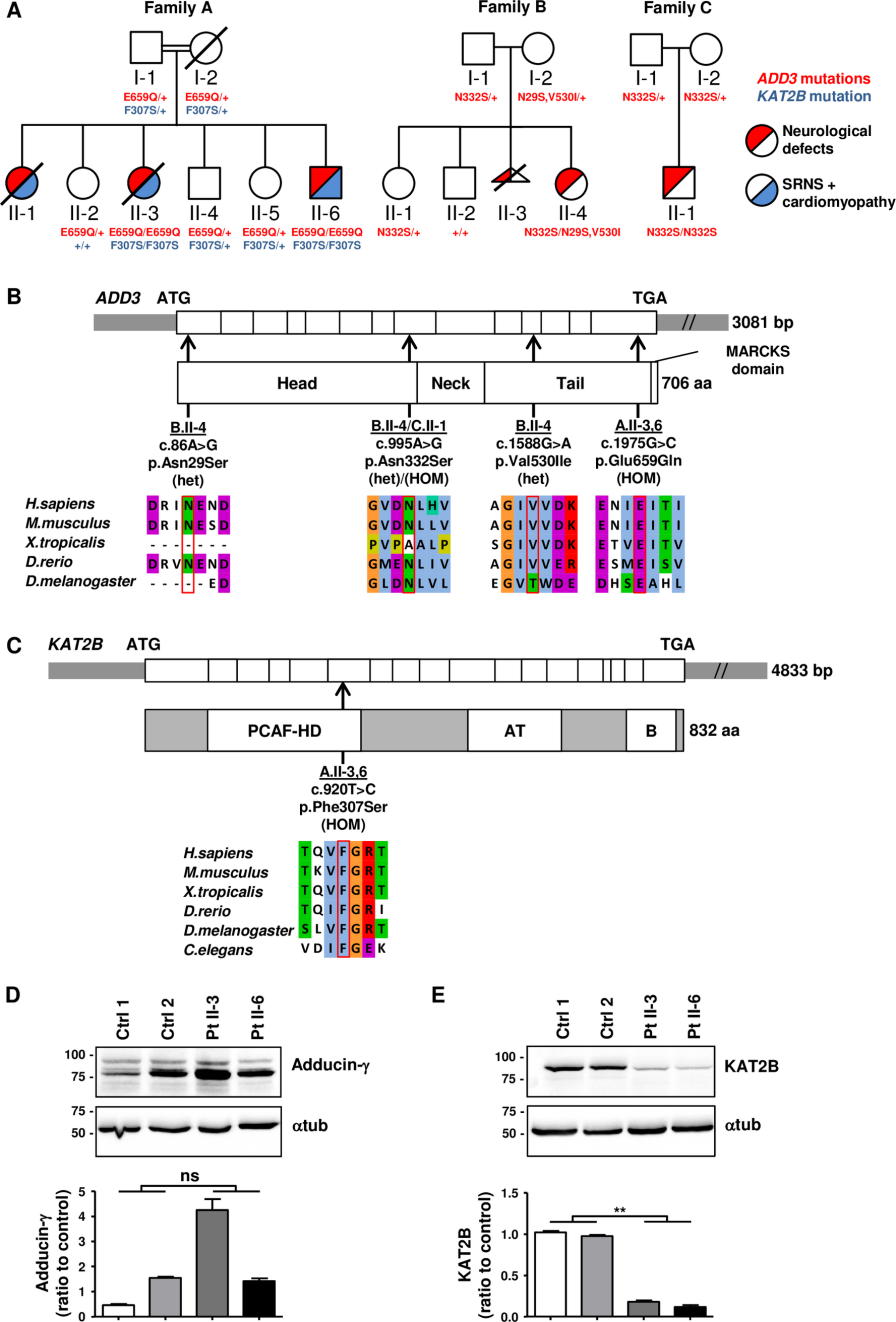
Identification of homozygous missense mutations in *ADD3* and *KAT2B* and effect of *ADD3* and *KAT2B* mutations on protein levels in fibroblasts. (A) Pedigree and segregation status of mutations found in *ADD3* and *KAT2B*. Discovery of *ADD3* mutations in family B and C was facilitated by GeneMatcher [44]. Half red coloured circles or squares denote patients with neurological defects and half blue coloured symbols denote patients with SRNS and cardiomyopathy. + symbols indicate non-mutated alleles. Mutations and segregation were confirmed by Sanger sequencing. (B) Exon structure of human *ADD3* cDNA (long isoform NP_058432) and domains of adducin-γ protein. The relative position of *ADD3* mutations to protein domains and exons are indicated (arrows). All mutations also affect the short isoform of *ADD3* (NP_001112). Below each mutation, the phylogenetic conservation of the altered amino acid residues is shown. (C) Exon structure of human *KAT2B* cDNA and domains of KAT2B protein. PCAF-HD, p300/CBP-associated factor homology domain; AT, acetyl transferase domain; B, Bromo domain. The relative position of *KAT2B* variation to protein domains and exons is indicated (arrow). The phylogenetic conservation of the altered amino acid residue is shown. (D, E) Adducin-γ (D) and KAT2B (E) protein levels in control and patient fibroblasts. Lysates of patient II-3 and II-6 (family A) fibroblasts and age-matched control fibroblasts (Ctrl 1 and 2) were analyzed by western blotting. Results were normalized to the loading control α-tubulin. Each quantification is shown in the lower panel (n = 3 independent experiments, student’s t-test).

For the affected individuals in family A and C, the presence of mitochondrial disease was excluded by muscle biopsy. Moreover, high-resolution karyotypes were normal for all patients, and CGH arrays (performed for family B and C) did not show significant abnormalities. Consequently, whole exome sequencing (WES) was performed on two affected members of family A as well as on the affected individuals and the parents of family B and C, after obtaining written informed consent and study approval. WES led to the identification of recessive potentially damaging mutations in *ADD3*, all segregating with the disease as confirmed by Sanger sequencing (*NM_016824*.*4*: family A: homozygous c.1975G>C, p.E659Q; family B: compound heterozygous c.86A>G, p.N29S; c.1588G>A, p.V530I (both on the same allele in the mother), c.995A>G, p.N332S (heterozygous in the father); family C: homozygous c.995A>G, p.N332S) (Fig 2A and 2B and Table 2). In 148,632 reference individuals from the gnomAD browser (http://gnomad.broadinstitute.org/), the *ADD3* mutations were present at low frequencies and only in the heterozygous state (Table 2).

**Table 2:** Pathogenic genetic variants identified in affected individuals with overlapping syndromes.

Family/ Individual	Gene	Nucleotide change	Amino acid change	Zygosity, Segregation	MT	SIFT	PolyPhen-2	gnomAD allele frequencies
**A/II-3, II-6**	*ADD3*	c.1975G>C	p.E659Q	HOM	DC	0.13 (T)	0.980 (PD)	4/246110 (no HOM)
	*KAT2B*	c.920T>C	p.F307S	HOM	DC	0 (D)	0.990 (PD)	Not reported
**B/II-3, II-4**	*ADD3*	c.86A>G	p.N29S	het m	DC	0.25 (T)	0.653 (PoD)	17/276960 (no HOM)
		c.995A>G	p.N332S	het p	DC	0.03 (D)	0.995 (PD)	176/276966 (no HOM)
		c.1588G>A	p.V530I	het m	DC	0 (D)	1 (PD)	9/276822 (no HOM)
**C/II-1**	*ADD3*	c.995A>G	p.N332S	HOM	DC	0 (D)	0.995 (PD)	176/276966 (no HOM)

Abbreviations are as follows: D, deleterious; DC, disease causing; het, heterozygous; HOM, homozygous; m, maternal; MT, mutationtaster; p, paternal; PD, probably damaging; PoD, possibly damaging; T, tolerated

The identified *ADD3* mutations result in the substitution of amino acids located in the head and tail region of the protein product adducin-γ (Fig 2B). In humans, adducins form heterotetramers that are composed of either adducin-α and -γ (the most widely expressed) or adducin-α and -β (restricted mainly to erythrocytes and specific brain regions) [9]. These heterotetramers regulate the actin cytoskeleton by capping the barbed ends of F-actin and by promoting the interaction between actin and spectrin [9, 10]. Recently, a homozygous mutation in *ADD3* was shown to cause cerebral palsy, epilepsy, borderline microcephaly, thin corpus callosum and intellectual disability in one family [7]. As the phenotype of this family shows overlap with all our families, particularly family C, our study supports the pathogenicity of the previously identified *ADD3* mutation.

### A homozygous variant in *KAT2B* associates with the extended phenotypes in family A

Family A, which was characterized by additional cardiomyopathy and SRNS, exhibited another potentially damaging homozygous mutation in lysine acetyltransferase 2B (*KAT2B)*. No other pathogenic variant was identifying after applying a set of filters excluding common variants in the general population (dbsnp>1%) or in our in-house database as well as variants predicted not to be deleterious. The *KAT2B* variant (*NM_003884*.*4*: c.920T>C, p.F307S) segregated with the disease and was not present in the reference individuals from the gnomAD browser (Fig 2A and Table 2). KAT2B is known to acetylate a variety of substrates, including histones (preferentially H3), and to function as a transcription coactivator together with CBP/p300 [11–13]. The identified *KAT2B* missense variant affects a highly conserved amino acid within the PCAF homology domain (Fig 2C), which is required for the interaction with CBP/p300 [14].

By studying mRNA and protein expression in patient fibroblasts from affected members of family A using qPCR, western blotting and immunostainings, we found no significant decrease for adducin-γ at the mRNA or protein level (Fig 2D and S1A and S1C Fig). However, KAT2B protein (but not mRNA) levels were significantly reduced (Fig 2 and S1B and S1D Fig). Thus, we reasoned that the *KAT2B* variant could contribute to the extended phenotype observed in family A. To test this hypothesis, we decided to perform functional validation of both mutations in *Drosophila melanogaster*.

### Adducin-*γ* E659Q is a hypomorphic mutation in *Drosophila*

**Fig 3:**
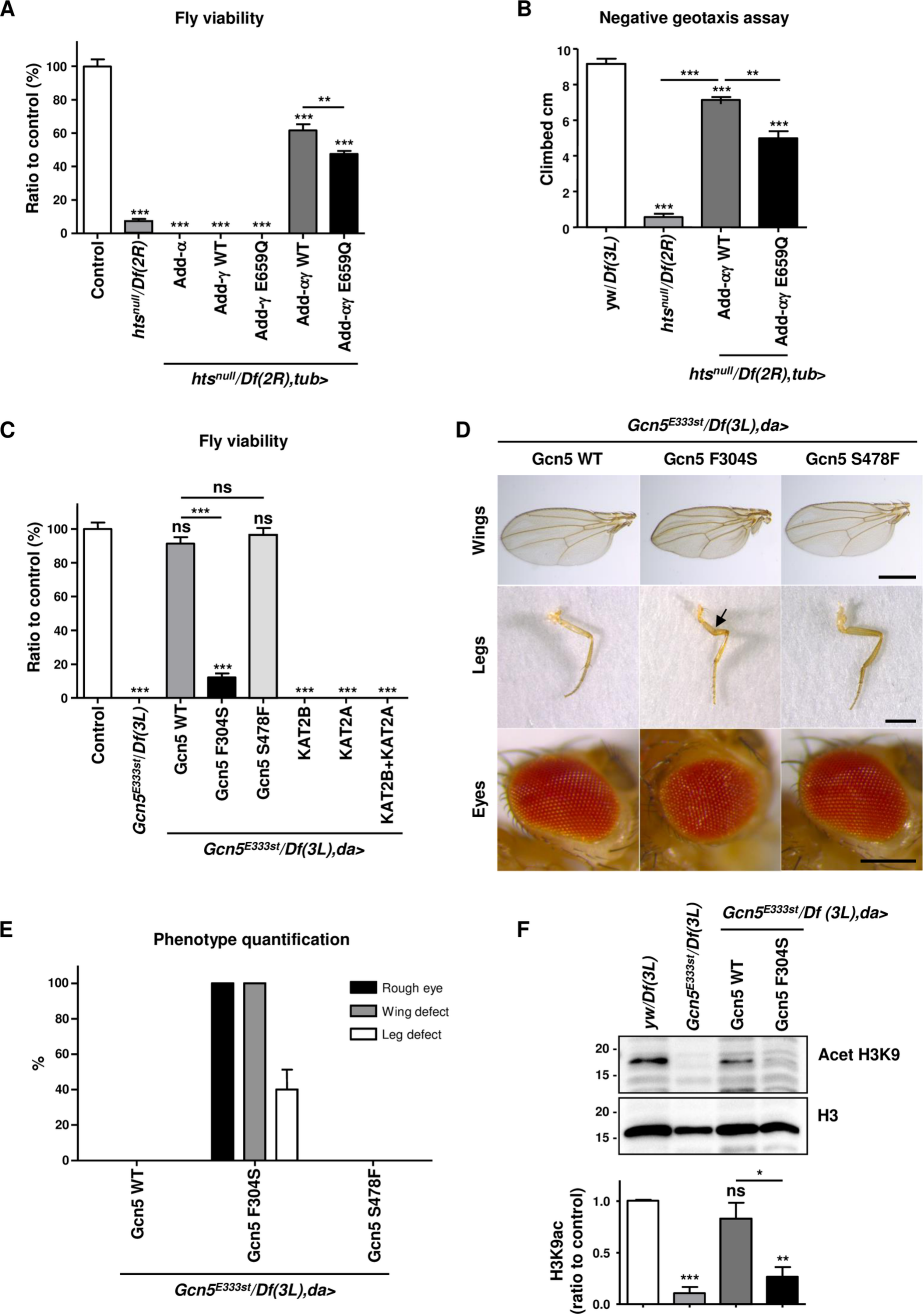
Effect of *ADD3* and *KAT2B* mutations on viability and morphology in *Drosophila*. (A) Viability for *hts*^*null*^ hemizygous flies and respective rescues with adducin (Add) construct(s) using *tubulin-*GAL4 (*tub>*). After 48h of egg laying on standard cornmeal/yeast food, viability was calculated as the percentage of hatching adults of the indicated genotype and normalized to the control. The control corresponds to the viable F1 trans-heterozygous flies obtained from the cross between *Df(2R)BSC26* (harbouring the *hts* gene) and a non-overlapping deficiency on the same chromosome (*Df(2R)247*). Quantification is for >100 F1 eclosing flies/genotype/experiment in 5 independent experiments. Statistical analysis was performed using one-way ANOVA with Bonferroni post-test. (B) Negative geotaxis assay for one-day-old adult flies. Flies were transferred to a graduated tube, and after tapping, the length climbed in 8 sec was recorded [45]. Quantification was performed on 6 independent experiments with >38 flies/genotype using one-way ANOVA with Kruskal-Wallis post-test. ns, non significant (see S1 Table for details on transgenic flies). For all panels: ns, non significant, *p<0.05 **p<0.01, ***p<0.001 (see S1 Table for details on transgenic flies). (C) Viability for *Gcn5*^*null*^ hemizygous flies and respective rescues with Gcn5 and KAT2A/B construct(s) using *daughterless-*GAL4 (*da>*). Viability was assessed as described in (A). Human *KAT2B* F307S and S502F mutations correspond to *Gcn5* F304S and S478F mutations, respectively. Gcn5 S502F variant predicted to be deleterious (PolyPhen-2 score of 0.98) was found on a healthy individual at the homozygous state in our in-house exome database. The control corresponds to the viable F1 trans-heterozygous flies obtained from the cross between *Df(3L)sex204* (harbouring the *Gcn5* gene) and a non-overlapping lethal mutant on the same chromosome (*CG3103*^*0MI0010*^). Quantification is for >100 F1 eclosing flies/genotype/experiment in 5 independent experiments. Statistical analysis was performed using one-way ANOVA with Bonferroni post-test. (D) Phenotype of Gcn5 WT, Gcn5 F304S and Gcn5 S478F rescue animals. Pictures correspond to adult flies one day post-eclosion and are representative of the defects found in wings (separated wing blades), legs (femur kinking, arrow) and eye (small and mild rough eye). Scale bars: wings: 500μm, legs: 500 μm, eye: 200μm. (E) Quantification of the defects in wings, legs and eyes found in Gcn5 F304S rescue flies is for >100 F1 eclosing flies/genotype. (F) H3K9 acetylation levels of *Gcn5*^*null*^ and Gcn5 WT and F304S rescue animals. Extracted nuclear proteins from 3^rd^ instar (= late) larvae were analysed by western blotting normalized to non-acetylated Histone 3 (H3). Quantification is shown in the lower panel (n = 3 independent experiments; one-way ANOVA with Bonferroni post-test). For all panels: ns, non significant, *p<0.05 **p<0.01, ***p<0.001 (see S1 Table for details on transgenic flies).

*Drosophila hu li tai shao (hts)* corresponds to the sole homolog of all three adducin genes in humans. As previously described [15], *hts*^*null*^ hemizygous animals died at the late larval stage, with only a few escapers progressing into adult stage. The escapers showed rough eyes, uncoordinated movements and inability to fly leading to death within 24h after eclosion (S2A Fig). For mutation validation, we re-expressed in this *hts*^*null*^ background the human wild-type (WT) and mutant constructs using the ubiquitous driver *tubulin (tub)*-GAL4 (see S1 Table for precise genotypes). As E659, the amino acid mutated in adducin-γ, is located in a very poorly conserved region (Fig 2B), we performed rescue experiments with WT and mutated human adducin-γ. While re-expressing each of the adducins alone failed to rescue the viability, the co-expression of adducin-α and -γ (hereafter referred to as adducin-αγ WT) led to around sixty percent of viable mutant adults (Fig 3A). Importantly, when co-expressing adducin-γ E659Q together with adducin-α (adducin-αγ E659Q), we observed a significantly reduced partial rescue of fly viability (Fig 3A). The surviving animals did not present with any defects in eye and wing morphology (S2A Fig) but showed climbing impairment in a geotaxis assay (Fig 3B) [7]. To express the transgenes with endogenous expression levels, we also used an available GAL4 insertion in the *hts* locus. This insertion leads to a partial lethality over *hts*^*nul*l^, which could be fully restored by adducin-αγ WT but not by E659Q (S3A Fig). Altogether, these results suggest that adducin-γ E659Q is a hypomorphic mutation.

### KAT2B F307S is a loss-of-function mutation in *Drosophila*

*Drosophila Gcn5* is homologous with *KAT2B* and its paralog *KAT2A*. *Gcn5*^*E333st*^ hemizygous animals died at late larval stage/early pupal stage as previously reported for this null mutation [16]. The expression of *Drosophila Gcn5* (hereafter referred to as Gcn5 WT) with *tub-*GAL4 or another ubiquitous driver (*daughterless (da)-*GAL4) led to a full rescue (S3B Fig and Fig 3C). By contrast, the expression of human *KAT2A* and *KAT2B*, either alone or in combination, did not restore the viability of the mutant (Fig 3C), suggesting that the human orthologs have evolved in structure and function in comparison to Gcn5. As the mutated amino acid in KAT2B, F307, is conserved in *Drosophila* Gcn5 (corresponding to Gcn5 F304), we re-expressed Gcn5 F304S in the *Gcn5*^*E333st*^ hemizygous background (Gcn5 F304S). As a negative control, we re-expressed a predicted potentially damaging *KAT2B* variant (S502F corresponding to Gcn5 S478F) found in a homozygous state in a healthy individual from our in-house database. While Gcn5 S478F rescue animals were normal (Fig 3C and S3B Fig), Gcn5 F304S had a dramatically decreased viability with death occurring either in pupal stages or a few days after eclosion (Fig 3C). All adult escapers showed blistered wings, inability to fly and rough eyes and around 40 percent of the animals had defects in leg morphology (Fig 3D and 3E). Interestingly, this phenotype corresponds to what has previously been described for the deletion of the entire PCAF homology domain, where the mutation is localized [16]. In agreement with the proposed function of Gcn5 in histone acetylation [16], we further detected histone (H3K9) acetylation defects for Gcn5 F304S but not for Gcn5 WT and control animals, as assessed by immunoblotting of larval nuclear extracts (Fig 3F), suggesting that the mutation impairs the enzymatic activity of Gcn5. Altogether, the results suggest that KAT2B F307S is a loss-of-function mutation in *Drosophila*.

### KAT2B F307S but not ADD3 E659Q causes cardiac defects in *Drosophila*

**Fig 4:**
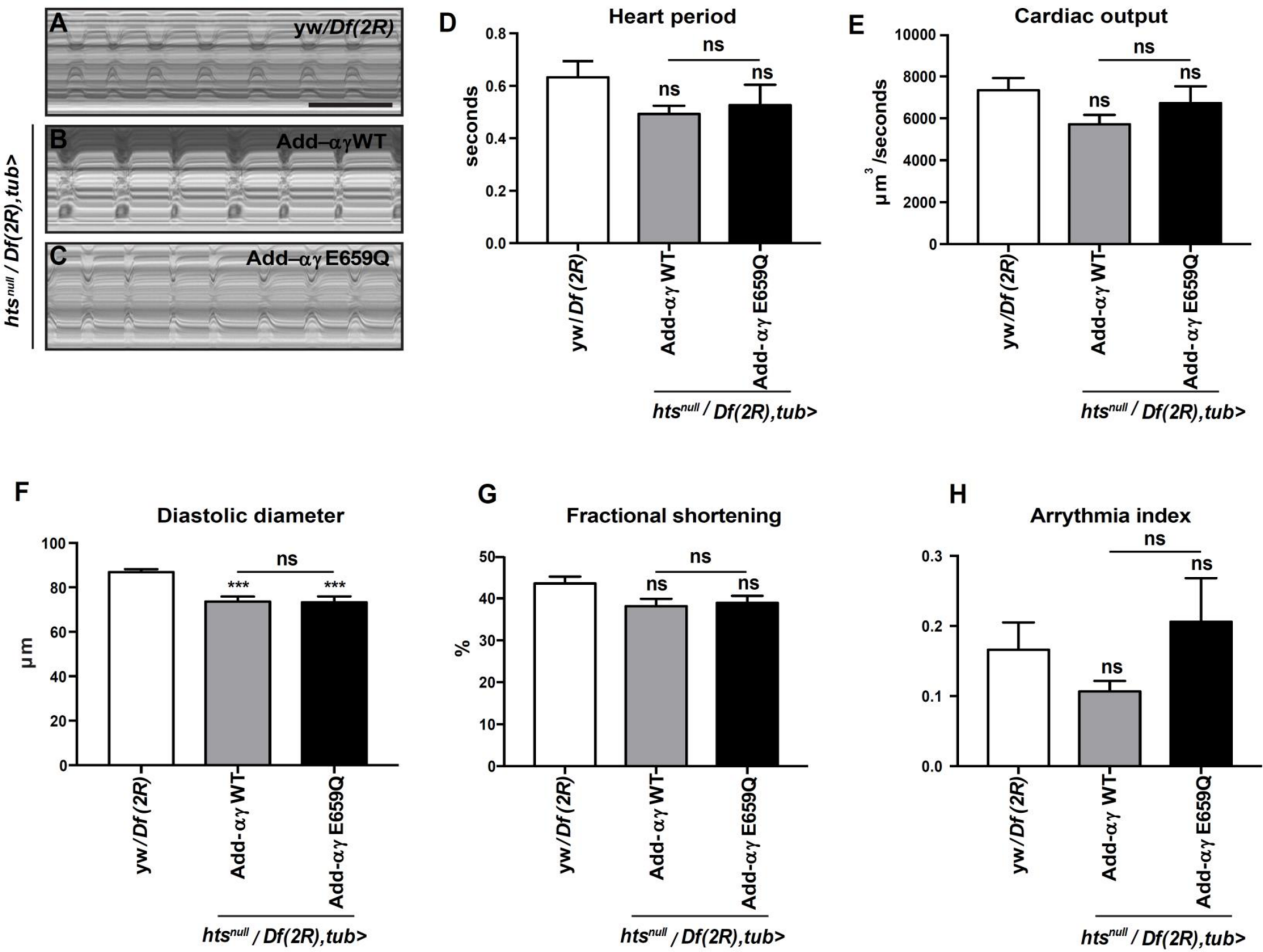
Effect of Gcn5 F304S mutation on *Drosophila* heart function. (A-C) M-mode kymographs of 1 day old beating hearts of control flies (*yw/Df(3L)*; A) and Gcn5^*null*^ flies rescued with Gcn5 WT (B) or Gcn5 F304S (C). Scale bar: 1 second. (D-H) High-speed movies of beating hearts were analysed using semi-automated Optical Heartbeat Analysis [46]. For quantification, 8–19 flies were analyzed. Statistical analysis was performed using one-way ANOVA and Tukey’s multiple comparison for all parameters except arrhythmia index (H), which was analysed using Mann-Whitney-Wilcoxon. For all panels: ns, non significant, *p<0.05 **p<0.01, ***p<0.001, ****p<0.0001 (see S1 Table for details on transgenic flies).

**Fig 5:**
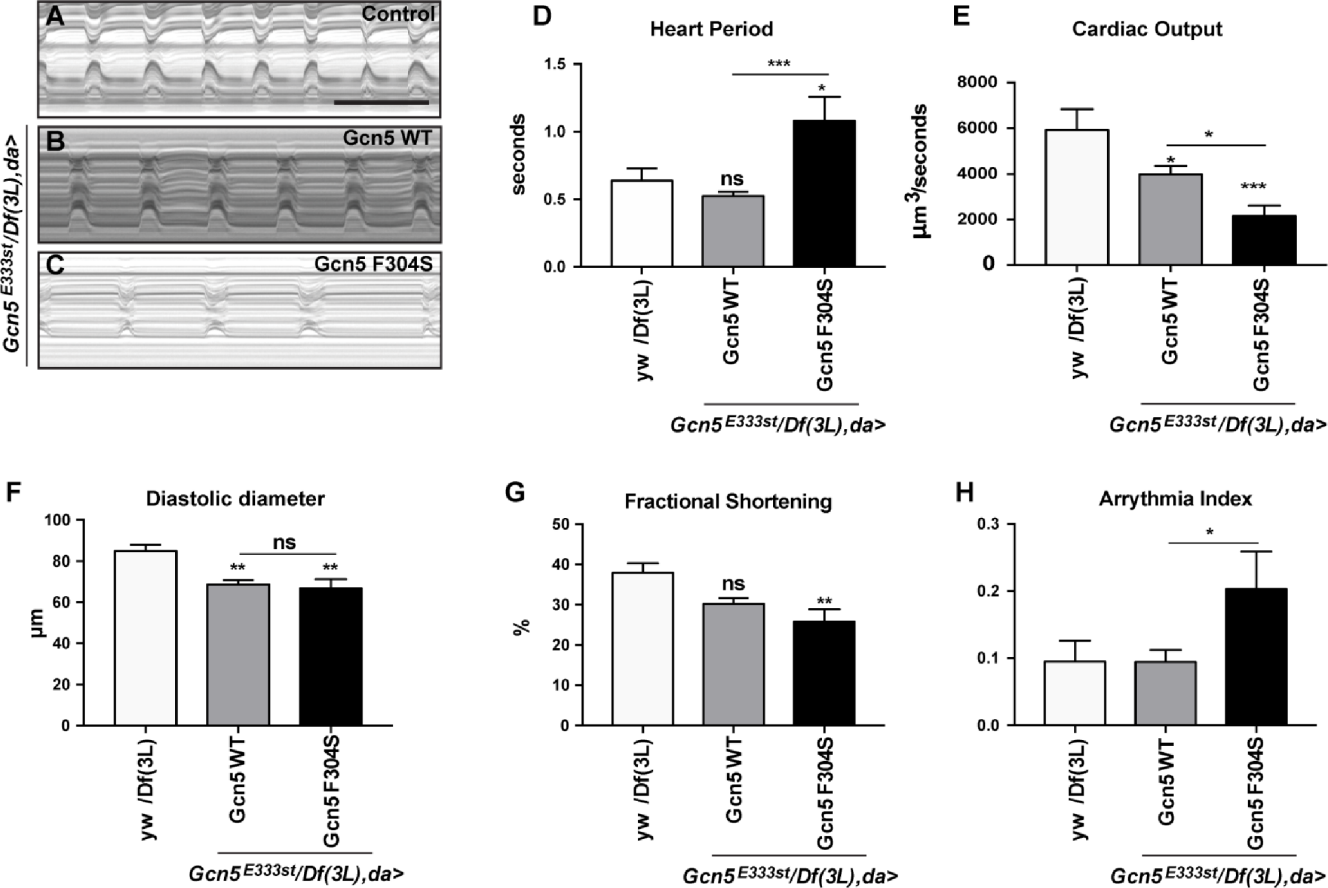
Effect of adducin-αγ E559Q on *Drosophila* heart function. (A-C) M-mode of beating 2-week-old control (*yw/Df(2R);* A), adducin-αγ WT (B) and adducin-αγ E559Q (C) rescue hearts. Scale bar: 1 second. (D-H) High-speed movies of beating adducin-αγ WT, adducin-αγ E559Q rescue and control hearts were analysed using semi-automated Optical Heartbeat Analysis [46]. For quantification, 8–19 flies were analyzed. Statistical analysis was performed using one-way ANOVA and Tukey’s multiple comparison, except for Arrhythmia index (H; n = 8–19, Mann-Whitney-Wilcoxon). For all panels: ns, non significant, ***p<0.001 (See S1 Table for details on transgenic flies).

Since the presence of SRNS and heart defects in family A was the main phenotypic difference from the other families, we looked more specifically into the cardiac and renal system of the fly. The *Drosophila* heart is a tubular organ formed by contractile cardiomyocytes that pump the hemolymph (analogous to the blood in vertebrates) to the rest of the body. This organ system has proven to be an important tool for studying the genetics and pathophysiology of cardiac disease [17, 18]. Therefore, we studied heart function in adult adducin and Gcn5 rescue flies. As illustrated in the M-mode traces obtained from high-speed movies, adducin-αγ E659Q did not show any significant differences in heart period, cardiac output, fractional shortening and arrhythmia index when compared to adducin-αγ WT (Fig 4). By contrast, Gcn5 F304S flies showed prolonged heart period and reduced cardiac output compared to Gcn5 WT and control flies (Fig 5A–5E). Both Gcn5 WT and F304S rescue flies showed a reduction in the normal diastolic diameter compared to control flies (Fig 5F), but only for Gcn5 F304S there was a reduction in contractility, measured as fractional shortening (Fig 4G). Moreover, the Gcn5 F304S mutant showed a more irregular heartbeat compared to Gcn5 WT, reflected by an increase in the arrhythmia index (Fig 5H). In further support of Gcn5’s requirement for normal heart function, the silencing of *Gcn5* with a heart-specific driver (*tin>*GAL4) led to a decreased cardiac output, an increased arrhythmia index and shortened diastolic diameter (S4B–S4D Fig). Interestingly, while the knockdown of *hts* did not cause any significant heart phenotypes (S4A–S4F Fig), the co-expression of *hts*^*RNAi*^ and *Gcn5*^*RNAi*^ significantly aggravated the heart period length and the arrhythmia index observed upon single knockdown of *Gcn5* (S4A and S4C Fig). The silencing efficiency for both RNAi lines were confirmed by qPCR and immunocytochemistry (S5A–S5D Fig). Altogether, the results suggest that Gcn5 is important for heart function in *Drosophila and* that Hts deficiency can increase the phenotypic consequences of *Gcn5* knockdown.

### KAT2B F307S but not adducin-γ E659Q causes renal defects in *Drosophila*

**Fig 6:**
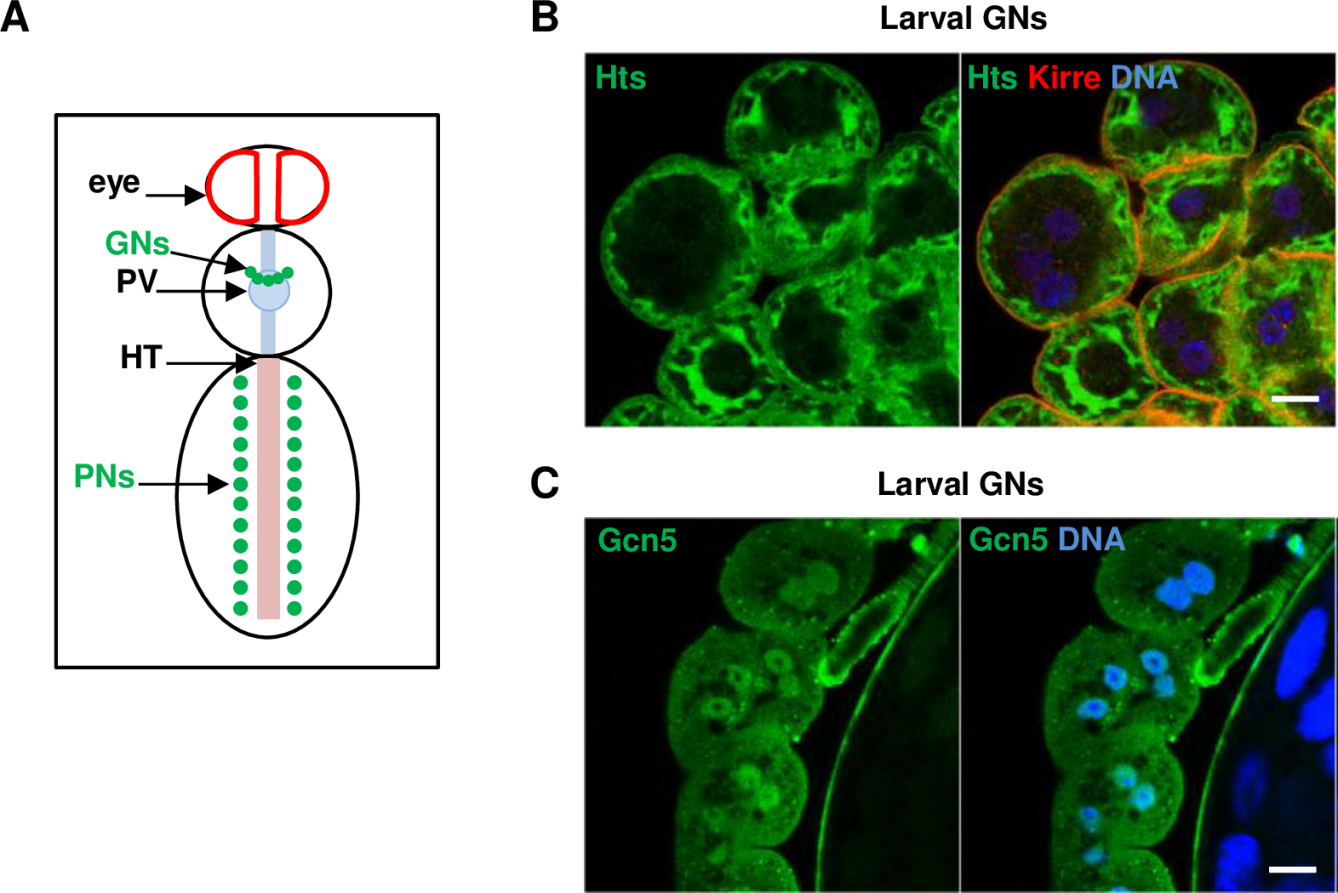
Expression and localization of Hts and Gcn5 in garland nephrocytes. (A) Schematic drawing of the localization of garland nephrocytes (GNs) and pericardial nephrocytes (PNs). The garland cells are attached to the proventriculus (PV) whereas the pericardial nephrocytes are lining the heart tube (HT). (B, C) Dissected wild-type (WT) garland nephrocytes were stained for Hts (B; green) and Gcn5 (C; green). At the time of dissection, larvae were in the third instar stage (the same for all other garland nephrocyte stainings). Kirre is in red (B). Nuclei were stained with Hoechst (B,C; blue). Scale bars: 10 μm.

The fly kidney is composed of garland and pericardial nephrocytes (Fig 6A) that perform the filtration of the hemolymph and Malpighian tubules that function as excretory tubes. The surface of nephrocytes is decorated with actin-anchored slit diaphragms showing high molecular similarity with those of mammalian podocytes [19–21]. Therefore, nephrocytes have successfully been used to functionally validate candidate genes for SRNS [22, 23].

By immunostaining, we observed that endogenous Hts localizes below the slit diaphragms at the cell cortex of larval garland nephrocytes (Fig 6B). A similar localization pattern was found when adducin-γ was overexpressed with its binding partner adducin-α. In this case, adducin-γ protein levels were significantly increased compared to expressing adducin-γ alone, suggesting that the stabilization by adducin-α is a prerequisite for proper function (S6 Fig). For Gcn5, we found a prominent expression in nephrocyte and podocyte nuclei (Fig 6C). The endogenous localization patterns were specific as they were lost upon *hts* and *Gcn5* knockdown, respectively (S5A and S5B Fig).

**Fig 7:**
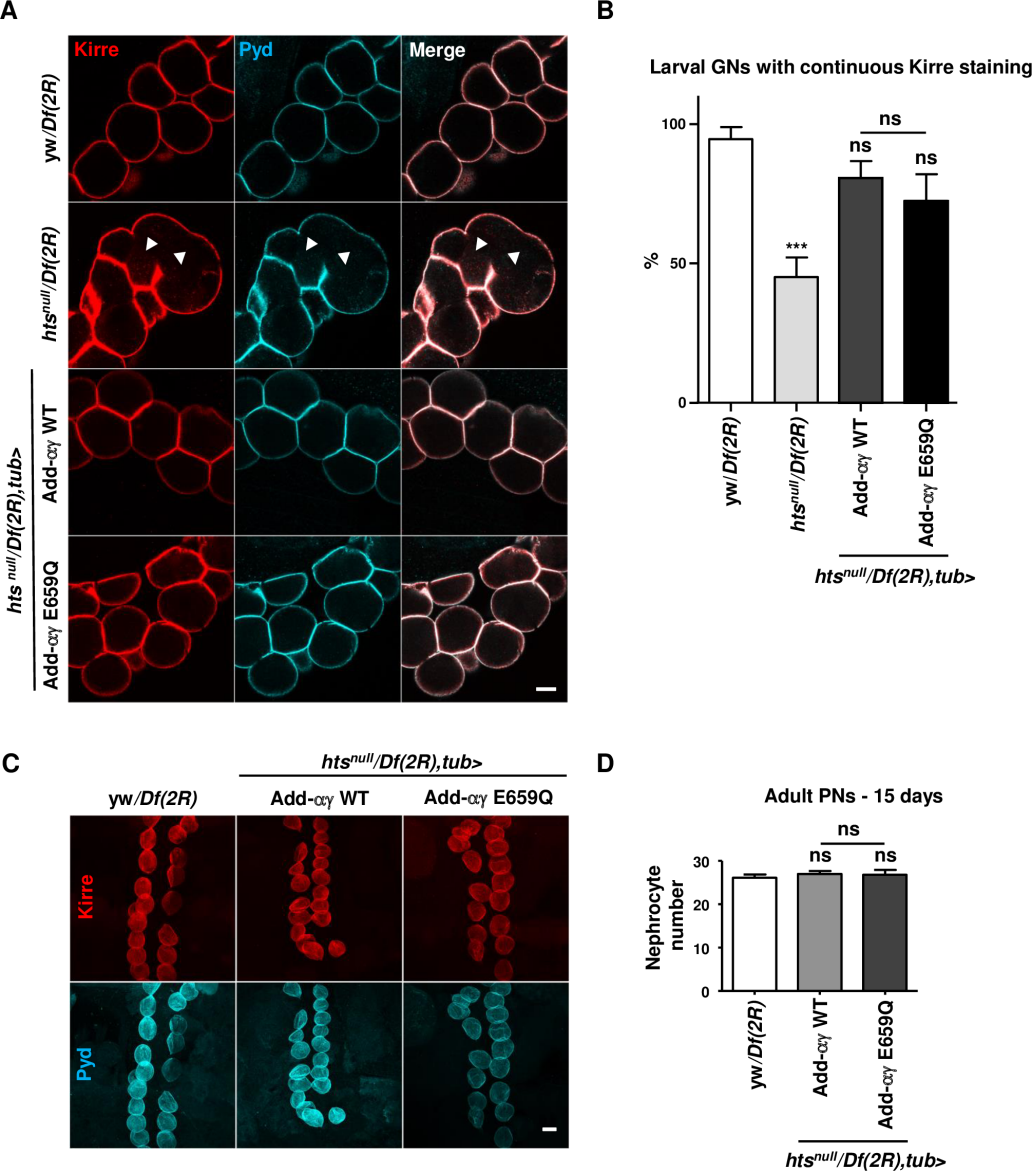
Garland nephrocyte phenotype of *hts*^*null*^ and adducin-αγ rescue mutants. (A) Kirre and Pyd localization in *hts*^*null*^ and rescue mutant garland nephrocytes. Dissected nephrocytes of the indicated genotypes were stained for Kirre (red) and Pyd, corresponding to Neph1 and ZO-1 in vertebrates, (blue). Arrowheads show areas of cell fusion. Scale bar: 10μm. (B) Quantification of nephrocytes showing a continuous Kirre staining using >9 samples/genotype from 3 independent experiments. Statistical analysis was performed with Kruskal-Wallis with Dunn’s post-test. ns, non significant, *p<0.05, ***p<0.001 (see S1 Table for details on transgenic flies). (C) Pericardial nephrocytes in adducin-αγ WT and E559Q rescue and control adult flies at 15 days post-eclosion were stained for the differentiation markers Kirre (red) and Pyd (blue). Note that *hts*^*null*^ is lethal at this stage. Scale bar: 30μm. (D) Quantification of the number of pericardial nephrocytes from n>8 samples/genotype in 3 independent experiments. Statistical analysis was performed using one-way ANOVA with Bonferroni’s post-test. ns, non significant (See S1 Table for details on transgenic flies).

To study the requirements of Hts for the integrity of the slit diaphragm, we performed immunostainings for Kirre, the ortholog of the mammalian slit diaphragm protein Neph1 [24]. In line with the proposed role for adducin-γ in cortical actin regulation [25], we found that *hts*^*null*^ larval garland nephrocytes showed a decrease of Kirre between adjacent nephrocytes (Fig 7A and 7B). When rescued with adducin-αγ WT and E659Q transgenes, however, no major differences with respect to Kirre localization were seen in larval garland nephrocytes (Fig 7A and 7B). Similarly, the morphology and number of adult pericardial nephrocytes were normal in both rescue animals compared to the control (Fig 7C and 7D).

**Fig 8:**
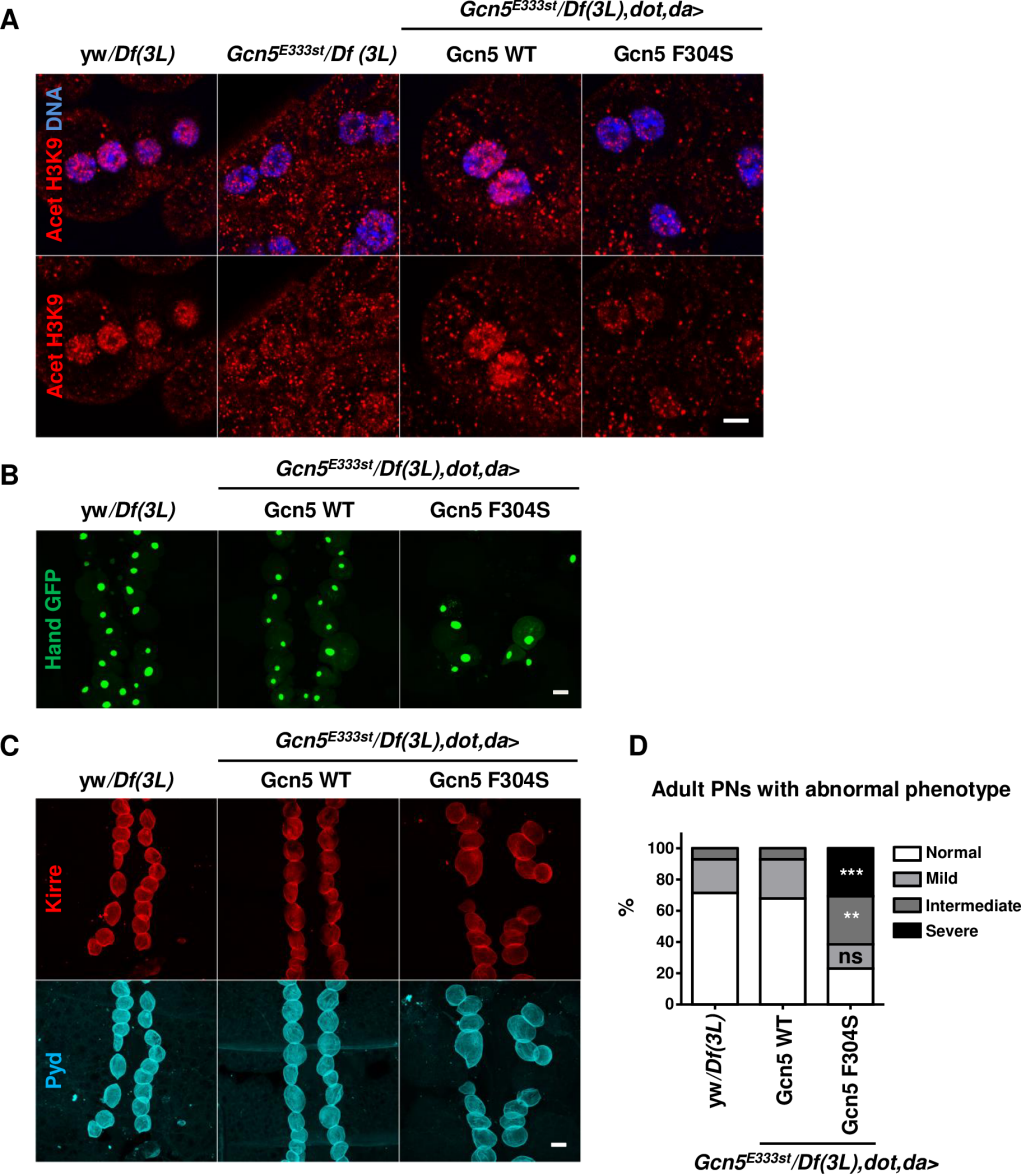
Effect of *Gcn5*/*KAT2B* variant on histone acetylation and survival of *Drosophila* nephrocytes. (A) Acetylated H3K9 in larval garland nephrocytes of *Gcn5*^*null*^ and Gcn5 WT and mutant rescue animals. *Dorothy (Dot)-*GAL4 (a nephrocyte specific driver) is used in combination with *da-*GAL4 as the latter shows only minor expression in nephrocytes. Garland nephrocytes of the indicated genotypes were stained for acetylated H3K9 (red) and Hoechst (blue). Scale bar: 5 μm. (B) Pericardial nephrocytes in adult *Gcn5* rescue mutant flies (7–15 days after eclosion) that express transgenic GFP (green) driven under the *Hand* promoter (*Hand*-GFP), specific for nephrocytes and cardiomyoblasts. Dissected pericardial nephrocytes were fixed with PFA and observed directly for GFP signal. Scale bar: 30 μm. (C) Pericardial nephrocytes in adult *Gcn5* rescue mutants (7–15 days after eclosion). Dissected pericardial nephrocytes of the indicated genotypes were stained for the differentiation markers Kirre (red) and Pyd (blue). Scale bar: 30 μm. (D) Quantification of the pericardial nephrocyte defects found in *Gcn5*^*null*^ rescue mutants (n>13/genotype; 3 independent experiments; Chi-square test). Nephrocytes with abnormal phenotypes included nephrocytes with abnormal distribution, abnormal shape, multinucleated or fragmented nuclei and reduced number of nephrocytes (<20). Phenotype severity was scored as normal (0), medium (1), intermediate (2) and severe (>2). For all panels: ns, non significant, **p<0.01, ***p<0.001 (see S1 Table for details on transgenic flies).

*Gcn5*^*E333st*^ hemizygous larvae presented with morphologically normal nephrocytes. Yet, we did detect a decreased H3K9 acetylation at this stage in the nephrocyte nuclei of the mutant, which could be rescued by Gcn5 WT but not by Gcn5 F304S (Fig 8A). Moreover, the majority of adult Gcn5 F304S escapers showed mislocalized and/or abnormally shaped pericardial nephrocytes in the adult stage that were often reduced in number (Fig 8B–8D), consistent with previously reported characterizations of important podocyte genes [26–28].

Together, the results demonstrate that, while Hts is important for nephrocyte function, the *ADD3* missense mutation identified in family A is alone insufficient to cause a renal phenotype in flies. By contrast, Gcn5 F304S seems to impair Gcn5 function in nephrocytes.

### Synergistic effects of hts and Gcn5 in *Drosophila* nephrocytes

**Fig 9:**
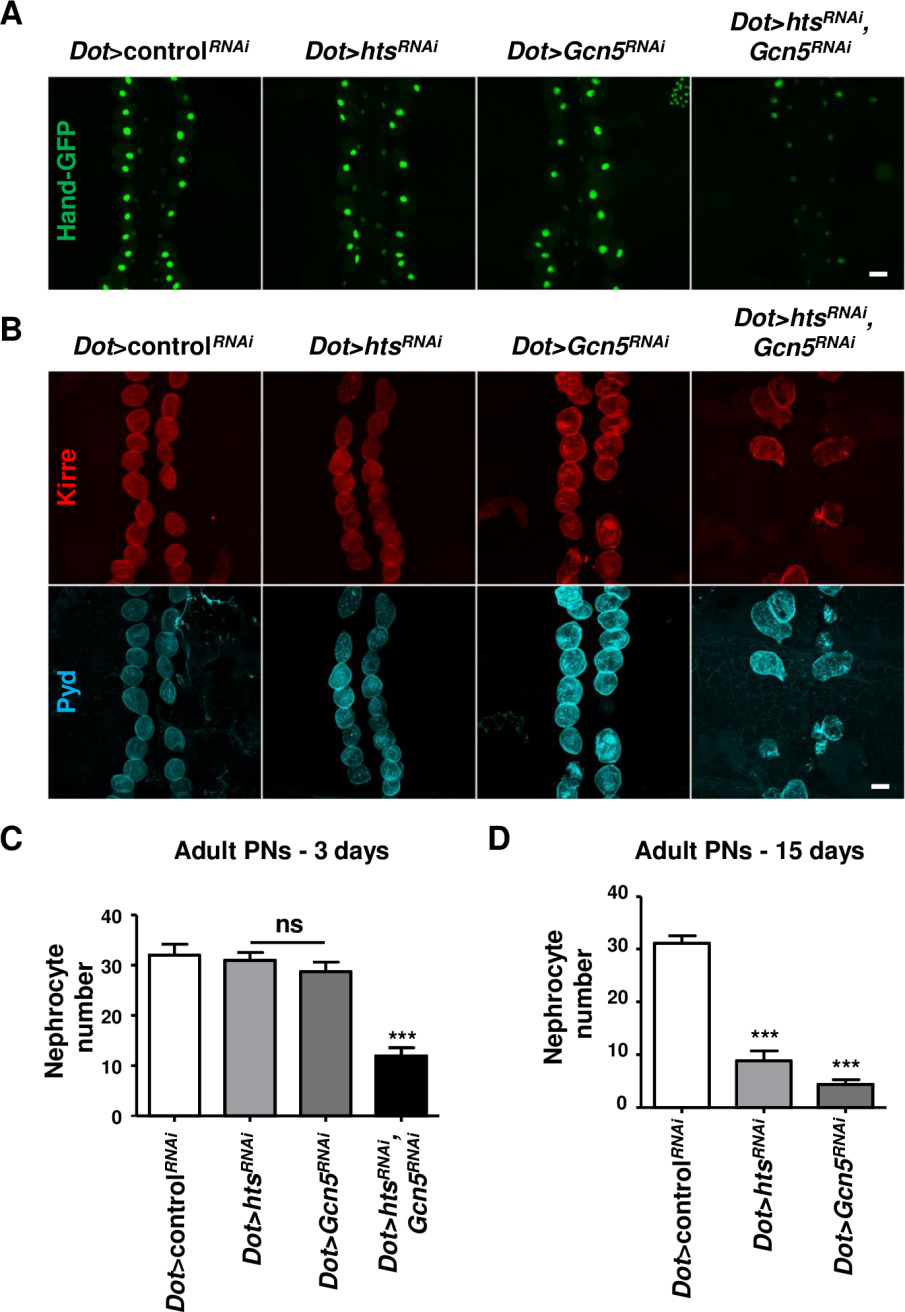
Number of pericardial nephrocytes in single and double knockdown of *hts* and *Gcn5*. (A-D) *Dot-GAL4*-mediated knockdown of *hts* or/and *Gcn5* in pericardial nephrocytes. Pericardial nephrocytes of adult flies with the Hand-GFP background were observed directly for GFP signal after fixation (A). Immunostaining was performed for the differentiation markers Kirre (red) and Pyd (blue; B). Images are representative of pericardial nephrocytes dissected from adult flies at 3 days post-eclosion. Scale bars: 30μm. Graphs represent quantification of the number of pericardial nephrocytes at 3 days (C) and 15 days post-eclosion (D) using >15 samples/genotype from 3 independent experiments. Statistical analysis was performed with Kruskal Wallis with Dunn’s post-test. For all panels: ns, non significant, ***p<0.001 (See S1 Table for details on transgenic flies).

To address any functional synergism between Gcn5 and Hts in nephrocytes, we performed nephrocyte-specific silencing of *Gcn5* and *hts* alone or in combination. In larval nephrocytes, the double knockdown of *Gcn5* and *hts* caused an increase in Kirre mislocalization, compared to the single knockdowns (S7A and S7B Fig). Moreover, while in 3-day-old adults *hts* knockdown did not affect pericardial nephrocyte number (Fig 9A–9C), a significant decline of differentiated nephrocytes could be observed in 15-day-old adults (Fig 9D). Similarly, the nephrocyte-specific expression of *Gcn5*^*RNAi*^ caused a progressive decline of differentiated pericardial nephrocytes at 15 days, but not at 3 days post eclosion (Fig 9A–9D). By contrast, the double knockdown of *Gcn5* and *hts* caused a significant loss of differentiated nephrocytes already at 3 days post-eclosion (Fig 9A–9C).

**Fig 10:**
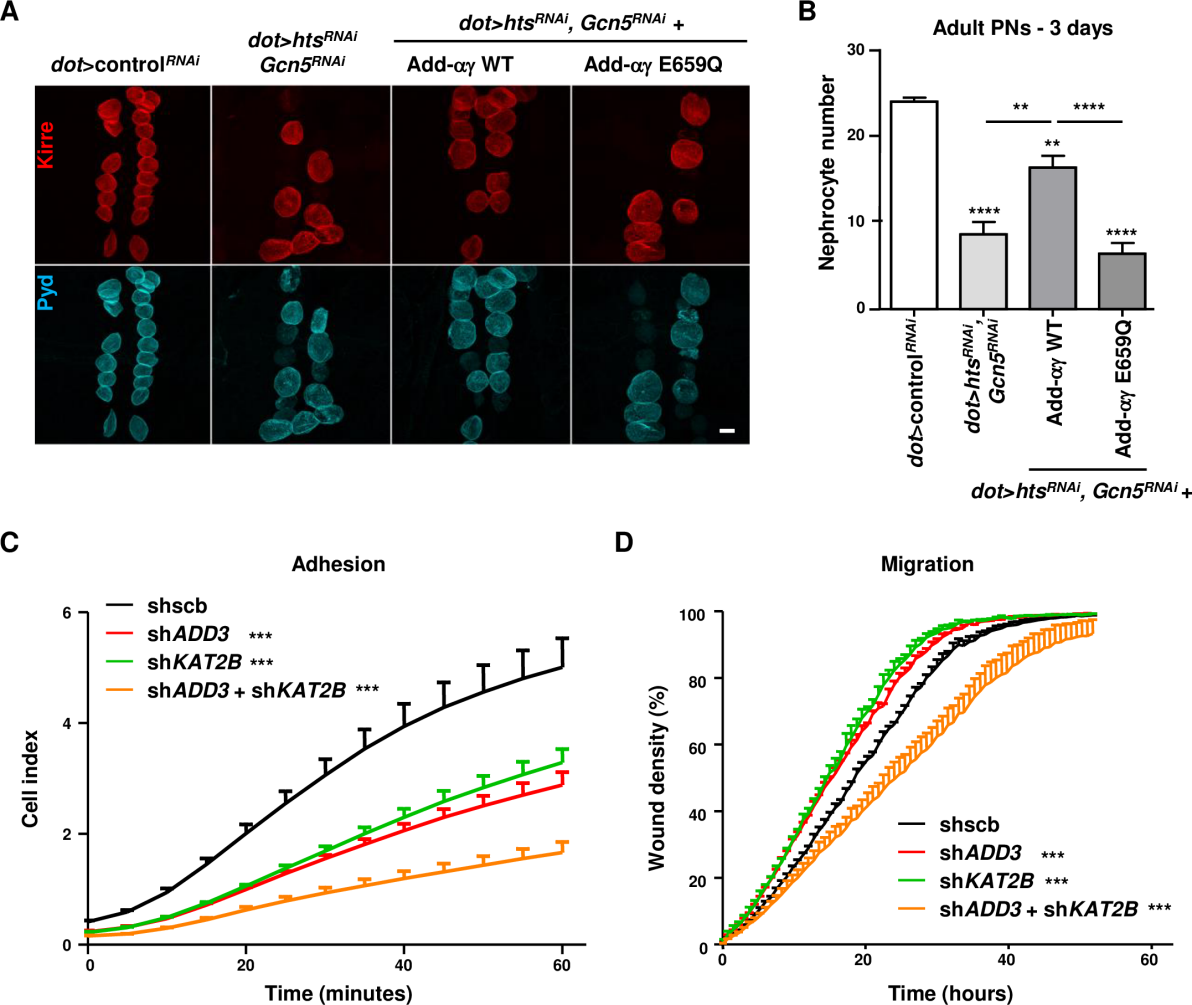
Synergism between ADD3 E659Q and Gcn5 knockdown in nephrocytes and effect of the double knockdown of ADD3 and KAT2B in human cultured podocytes. (A, B) WT or mutated adducin rescue constructs were expressed in *hts* and *Gcn5 double knockdown* pericardial nephrocytes to study the interaction between E659Q mutation and gcn5 loss of function. (A) Immunostaining was performed for Kirre (red) and Pyd (blue). Images are representative of pericardial nephrocytes dissected from adult flies at 3 days post-eclosion. Scale bar: 30μm. (B) Graphs represent quantification of the number of pericardial nephrocytes at the same time point using >20 samples/genotype from 3 independent experiments. Statistical analysis was performed with One-way ANOVA with Dunnett’s post-test. For all panels: **p<0.01, ***p<0.001, ****p<0.0001 (See S1 Table for details on transgenic flies). (C) Adhesion was assessed using the xCELLigence system (ACEA Biosciences). Cells were plated with complete medium in the E-plate 96. Data obtained were analyzed with the RTCA software. Results are presented as time *vs*. cell index curve (n = 3 independent experiments; linear regression analysis). (D) Migration was assessed using the Incucyte Scratch wound cell migration assay (Essen Bioscience). Cells were plated with complete medium 48 hours before scratch in ImageLock Plates-96 wells (Essen Bioscience) and images were recorded every 45 minutes after scratch until complete wound closure. Images were analyzed using the Incucyte Zoom software. Results are presented as percentage of wound cell density over time (n = 3 independent experiments; linear regression analysis). For all panels: **p<0.01, ***p<0.001, ****p<0.0001.

Making use of the double knockdown phenotype, we also addressed the synergism between *ADD3* and *KAT2B* mutations from family A. Considering that the *KAT2B* variant corresponds to an almost complete loss of function mutation, we performed a double knockdown of *Gcn5* and *hts* rescued only with the adducin-αγ transgenes, thereby avoiding the complexity of bringing all the *Gcn5* and *hts* alleles as well as the GAL4 driver and respective rescue constructs together in one fly. In this setting, the adducin-αγ WT combination partially rescued the loss of pericardial nephrocytes in 3-day old adult flies (Fig 10A and 10B). By contrast the expression of the adducin-αγ E659Q combination showed the same degree of nephrocyte loss as the double knockdown at this stage. Together, these results suggest a functional interaction between *KAT2B* and *ADD3* mutations in the nephrocyte, which may be of relevance for the renal phenotypes in family A.

### Synergistic effects of adducin-γ and KAT2B in human podocytes

To validate our findings in human cells, we studied adducin-γ and KAT2B in cultured human podocytes. While KAT2B was as expected found in nuclei of podocytes, adducin-γ localized to the cell periphery similar as in nephrocytes (S8A–S8D Fig). Both localization patterns were specific, as they were reduced upon lentiviral transduction of respective shRNAs and could be restored by re-expression of both wild-type and even mutant adducin-γ and KAT2B (S8A–S8D Fig).

To address the phenotypic effects of the *ADD3* and *KAT2B* knockdowns, we analyzed adhesion and migration, which are processes typically affected in kidney diseases such as SRNS [29, 30]. While podocyte adhesion was reduced in the single *ADD3* and *KAT2B* knockdowns, the double knockdown showed additive effects (Fig 10C). With regard to migration, the single knockdown showed a mildly increased migration. By contrast, the double knockdown led to a strongly impaired migration (Fig 10D), providing further evidence for potential synergistic effects of mutations in both genes in the kidney.

## Discussion

Here, we identify *ADD3* mutations in three different families with similar neurological, skeletal and ophthalmological phenotypes, thereby consolidating and expanding the mutational and phenotypic spectrum of *ADD3* deficiency reported initially. Moreover, we use functional validation in *Drosophila* and human cells to characterize the contribution of an additional variant in *KAT2B* to the extended phenotype featuring one of the *ADD3* families. While such additional variants are commonly excluded from WES datasets without even performing functional validation, particularly when the respective gene functions seem to be unrelated to the disease(s) in question, our results demonstrate that both the *ADD3* and the *KAT2B* mutation could be pathogenic. Moreover, we show that *ADD3-*associated phenotypes can be unmasked by additional Gcn5/KAT2B deficiency in nephrocytes and human podocytes. Our study thus provides an example of a genetic disease where the tissue manifestation could be influenced by a second homozygous mutation on another chromosome.

KAT2B has previously not been associated with any genetic disease. The severity of the *KAT2B* variation in the fly model compared with the relatively late-onset cardiac and renal defects in the patients indeed suggests a partial functional redundancy due to gene duplication in vertebrates. Accordingly, it has been shown that in mouse development loss of KAT2B can be compensated for by KAT2A [31]. Nevertheless, KAT2B is strongly expressed in mouse heart and kidney, particularly podocytes [32, 33], while KAT2A has a more widespread expression pattern [14]. Moreover, mouse and zebrafish studies have shown that KAT2B can perform functions that are non-redundant with KAT2A, even in the heart [34–39], indicating that *KAT2B* deficiency alone could be sufficient for any clinical manifestations. What remains to be seen is whether the clinical impact of *KAT2B* deficiency needs to be uncovered by a sensitized genetic background, such as the *ADD3* mutation, or whether it is the other way around. While our data do not exclude either possibility, it is interesting that rare but otherwise uncharacterized variants of *KAT2B* have been found to be enriched in a patient cohort with sporadic FSGS [32], suggesting that KAT2B could be a susceptibility factor for FSGS forms with different primary causes.

At the level of protein function and disease mechanism, the precise mode of interaction between adducin-γ and KAT2B is equally unclear. Apart from histones, KAT2B has recently been shown to acetylate a variety of proteins [40]. Among them are also cytoskeletal regulators, that when mutated cause FSGS or cardiomyopathy (e.g. actinin-4, TTC21B and myosin-7). Thus, it is possible that adducin-γ could also be a target of KAT2B-dependent acetylation. Vice versa, any influence of adducin-γ defciciency on the activity of KAT2B cannot be excluded. Apart from more mechanistic functional studies, the identification of more patients with the same or other variants in only *ADD3* or *KAT2B* combined with careful characterization of their phenotypes will be crucial to define the precise role of each gene and their potential functional interaction in humans.

## Materials and methods

### Ethical statement

Following informed written consent, we obtained clinical data, blood samples and skin biopsies from the affected individuals. This study was conducted with the approval of the Comité de Protection des Personnes pour la Recherche Biomédicale Ile de France II. Approval was obtained under the number DC 2014–2272

### Whole exome sequencing

Whole-exome sequencing (WES) was performed for affected individuals II-3 and II-6 from family A and for the two parents and the affected sib from family B and family C. Whole-exome capture was performed with the Agilent SureSelect Human All Exon Kit, 51Mb, V4 (family A), the Roche MedExome kit (family B) or a proprietary system from GeneDx (family C). The enriched library was then sequenced on either Life Technologies SOLID (paired end with 75+35 base pair (bp) reads; family A) or Illumina systems (family B: 2x150 bp reads; family C: 2x100bp read). Images were analyzed and the bases were determined according to Lifescope or bcl2fastq Conversion Software v2.17. Variants were called as described [41].

### Fly strains and generation of transgenic flies

Crosses were maintained on standard cornmeal-yeast food at 25°C except for RNAi crosses (29°C). The fly stocks were used in this study can be found in S1 Table. For *Hts*^*null*^ rescue constructs we used an N-terminal V5 tagged human *ADD3* (clone IMAGE: 6649991), WT or carrying the E659Q mutation, and the N-terminal HA tagged human *ADD1 (*gift from Vann Bennett, Duke University). For *Gcn5*^*null*^ rescue constructs we used the C-terminal HA tagged human *KAT2B* (clone IMAGE: 30333414), the N-terminal Flag tagged human *KAT2A* (gift from Laszlo Tora, Institut de Génétique et de Biologie Moléculaire et Cellulaire, Strasbourg) and the C-terminal Flag tagged *Drosophila Gcn5*, WT (gift from Clement Carré, University Pierre et Marie Curie) or carrying the mutations F304S or S478F (corresponding to human mutation F307S and S502F). All mutations were inserted using the QuickChange site-directed mutagenesis kit (Stratagene) according to the manufacturer’s protocol. Subsequently, the rescue constructs were subcloned into a *pUASTattB* vector (gift from *Konrad Basler*, University of Zurich) and injected into flies at *attP* landing sites by Bestgene, USA.

### Cell culture

A conditionally immortalized human podocyte cell line developed by transfection with the temperature-sensitive mutant (tsA58) of the SV40-T-antigen-encoding gene, was kindly provided by Dr. Saleem (University of Bristol). In brief, the cells proliferated at the permissive temperature of 33°C, whereas growth arrest and differentiation were induced by incubation at the nonpermissive temperature of 37°C for 14 days. Cells were grown with 7% CO_2_ in RPMI 1640 medium supplemented with 10% fetal bovine serum, insulin-transferrin-selenium, glutamine, penicillin and streptomycin (all from Life Technologies).

Primary skin fibroblasts were obtained from individual II-3 and II-6 from family A and two different age matched controls. These cells were grown in OPTIMEM medium supplemented with 20% fetal bovine serum, glutamine, penicillin and streptomycin (all from Life Technologies) at 37°C with 7% CO2.

### Establishment of lentiviral cell lines

Small hairpin RNAs (shRNAs) Scramble (Scb) or targeting the 3’UTR of human *ADD3* and *KAT2B* mRNA in the lentiviral vector pLKO.1 were purchased from Sigma (*ADD3* clone: NM_019903.3-2280s1c1 TRCN0000123024; *KAT2B* clone: NM_003884.4-3192s21c1, TRCN0000364135). Lentiviral particles containing these constructs were produced in human embryonic kidney 293T cells as previously described [42]. *ShScb*, *ADD3* or *KAT2B* depleted podocytes were obtained by transduction with the respective shRNAs lentiviral particles and subsequent puromycin selection.

Human *ADD3 and KAT2B*, were subcloned from human full-length cDNA (*ADD3*: clone IMAGE: 6649991; *KAT2B* clone IMAGE: 30333414) into the expression vectors pLentiGIII and PLEX-MCS, respectively. An HA tag was added in frame, before the stop codon, to the C terminus of *ADD3* and *KAT2B*. The *ADD3* E659Q and *KAT2B* F307S mutations found in affected individuals were introduced with the QuickChange site-directed mutagenesis kit (Stratagene) according to the manufacturer’s protocol. All constructs were verified by sequencing. *ADD3* or *KAT2B* depleted podocytes were transduced with WT or mutant *ADD3* or *KAT2B* lentiviral particles, respectively.

### RNA extraction, RT-PCR and real time quantification

Total RNA was isolated using a Qiagen RNA extraction kit (Qiagen), following the manufacturer’s instructions. cDNA was prepared using reverse transcriptase Superscript II (Invitrogen). PCR was performed using ReadyMix Taq PCR (Sigma). After RNA extraction and cDNA preparation by RT-PCR, relative expression levels of genes of interest were determined by real-time PCR using the Absolute SYBR Green ROX Mix (ABgene) and specific primers as follows: *ADD3* forward 5’-CTTGCTGGAATTGTTGTGGATAAG-3’ and reverse 5’-CTGGTGGGCCATGATCATC-3’; *KAT2B* forward 5’-ATCACACGGCTCGTCTTTGAC-3’ and reverse 5’-CACCAATAACACGGCCATCTT-3’; *hts* forward 5’-GCACTCCGGATCCCAAGAAG-3’ and reverse 5’-CAGGCACAAACTGGAGTGGA-3’, *Gcn5* forward 5’-CGATCGTCCAAGCAGTGAG-3’ and reverse 5’-TCCGCCTTGACGTTCTCATC-3’. Experiments were repeated at least three times and gene expression levels were normalized to human HPRT or *Drosophila melanogaster* actin.

### Immunoblotting

Total cell or third instar larvae total protein extractions were performed and the resolved proteins were probed using the primary antibodies: anti-PCAF rabbit monoclonal (3378, Cell Signaling,1:1000), anti-adducin-γ mouse monoclonal (sc-74474, Santa Cruz, 1:1000) and anti-αtubulin mouse monoclonal (T5168, Sigma Aldrich, 1:5000). For immunoblotting of nuclear extracts [43], the primary antibodies anti-acH3K9 rabbit polyclonal (39918, Active motif, 1:1000) and anti-H3 mouse monoclonal (61475, Active motif, 1:1000) were used as well as the corresponding HRP-conjugated secondary antibodies (Amersham ECL, GE healthcare and Invitrogen). Bands were visualized using Amersham ECL Western Blotting Detection Reagent (GE Healthcare) and quantified by densitometry using *Image J* software.

### Immunofluorescence

Fibroblasts or podocytes were plated on noncoated coverslips or coverslips coated with rat-tail collagen type I (Corning), respectively. After 48h of culture cells were fixed with 100% ice-cold ethanol. Cells were incubated with a blocking solution (PBS, 1% BSA, and 0.1% tween 20) and further permeabilized for ten minutes with PBS 0.1% Triton. Incubation with the following primary antibodies was done ON at 4°C: anti-PCAF mouse monoclonal (sc-13124, Santa Cruz, 1:100), anti-adducinγ rabbit polyclonal (sc-25733, Santa Cruz, 1:100) and anti-HA (11 867 423 001, Roche, 1:200).

For immunofluorescence in *Drosophila*, garland and pericardial nephrocytes were dissected from third instar larvae and adults, respectively, and fixed for 20 minutes in 4% paraformaldehyde at room temperature and stained according to standard procedures. For Kirre stainings an alternative fixation method (“heat fixation”) was used: nephrocytes were heat-fixed for 5 seconds at 90°C in 0.7% NaCl/0.05% TX-100 solution. The following primary antibodies were used: anti-Hts mouse monoclonal (#1B1 deposited to the Developmental Studies Hybridoma Bank (DSHB) by Lipshitz, H.D.), anti-Gcn5 rabbit polyclonal (gift from Jerry Workman, Stowers Institute for medical research, Kansas, 1:200) anti-Kirre rabbit polyclonal (gift from Karl Fischbach, Institute for Biology, Freiburg, Germany, 1:200), anti-Pyd2 mouse monoclonal (deposited to the DSHB by Fanning, A.S, 1:100), anti-acH3K9 rabbit polyclonal (#06-942, Upstate, 1:100), AlexaFluor488-conjugated anti-horseradish peroxidase (Jackson Immunoresearch, 1:400), anti-HA (11 867 423 001, Roche, 1:200) and anti-V5 rabbit polyclonal (v8137 Sigma, 1:200). The corresponding anti-isotype AlexaFluor antibodies (ThermoFisher Scientific, 1:200) were used at room temperature for 2 hours. Nuclei were stained with Hoechst. Confocal images were obtained with a Leica TCS-SP8 confocal microscope, and post-treatment analysis was performed with *Image J* software.

### Statistical analyses

Results are presented as means ± standard error or standard deviation for the indicated number of experiments. Statistical analysis of continuous data was performed with two-tailed Student *t* test for pairwise comparisons or one-way analysis of variance for comparisons involving three or more groups, with Dunnet’s, Bonferroni or Dunn post hoc test, as appropriate. Pearson’s chi-squared test was used for analysis of categorical data. Linear relations between variables were analysed using linear regression analysis. P<0.05 was considered statistically significant. Analysis was carried out with GraphPad Prism software. (*p<0.05; **p<0.01, ***p<0.001, ****p<0.0001). All experiments were performed at least three times.

## Supporting information

S1 Fig mRNA levels and subcellular localization of ADD3 and KAT2B in control and patient fibroblasts. (A, B) *ADD3* (A) and *KAT2B* (B) mRNA levels in patient fibroblasts were assessed by quantitative PCR. Experiments were repeated at least three times and gene expression levels were normalized to the house keeping gene *HPRT*. Statistical analysis was performed using student’s t-test; ns, non-significant. (C, D) Immunostaining was performed for adducin-γ (green; C) and KAT2B (green; D) in control and patient fibroblasts. Nuclei were stained with Hoechst (blue). Note the loss of nuclear staining for KAT2B in patient fibroblasts. Scale bars: 10 μm.
(TIF)

S2 Fig Morphology of *hts*^*null*^ flies and adducin-αγ WT or E659Q rescue mutants. Representative pictures of *hts*^*null*^ and respective adducin-αγ WT and E659Q rescue mutants one day post-eclosion. *hts*^*null*^ flies have rough eye and motor coordination defects and are unable to fly. The ubiquitous co-expression of adducin-α and -γ using *tub-*GAL4 rescues these defects regardless of the presence of the E659Q mutation. Scale bars: upper panel: 1mm, wings: 500μm, eye: 200μm.
(TIF)

S3 Fig Rescue experiments with alternative drivers. (A) Rescue of *hts* mutant viability defects with adducin-αγ transgenes driven by a GAL4 insertion in the endogenous *hts* locus. After 48h of egg laying on standard cornmeal/yeast food, viability was calculated as the percentage of eclosing adults of the indicated genotype and normalized to the control. The control corresponds to the viable F1 trans-heterozygous flies obtained from the cross between *Df(2R)BSC26* (harbouring the *hts* gene) and a non-overlapping deficiency on the same chromosome (*Df(2R)247*). The insertion of GAL4 in *hts* locus leads to partial lethality which is completely rescued by adducin-αγ WT but not by E659Q. Quantification is for >100 F1 eclosing flies/genotype/experiment in >5 independent experiments. Statistical analysis was performed using one-way ANOVA with Dunnett’s post-test. (B) Viability for *Gcn5*^*null*^ hemizygous flies and respective rescues using *tubulin-*GAL4 (*tub>*). Viability was assessed as described in (A). The control corresponds to the viable F1 trans-heterozygous flies obtained from the cross between *Df(3L)sex204* (harbouring the *Gcn5* gene) and a non-overlapping lethal mutant on the same chromosome (*CG3103*^*0MI0010*^). Quantification is for >100 F1 eclosing flies/genotype/experiment in >5 independent experiments. Statistical analysis was performed using one-way ANOVA with Bonferroni post-test. For all panels:ns, non significant, *p<0.05, **p<0.01, ***p<0.001, ****p<0.0001.
(TIF)

S4 Fig Effect of cardiac-specific depletion of *hts* and *Gcn5* on *Drosophila* heart function. (A-F) *Tin*-GAL4 driver was used to knockdown *hts* or/and *Gcn5* in cardiomyocytes, and different heart parameters were analyzed in 3 week-old adult flies. Two separate control RNAi lines (TRIP and KK) were used to match *Gcn5*^*RNAi*^ (TRIP) and *hts*^*RNAi*^ (KK), respectively. For quantification, 19–30 flies were analyzed. Statistical analysis was performed using one-way ANOVA and Tukey’s multiple comparison for all parameters except arrhythmia index, which was analysed using Mann-Whitney-Wilcoxon. For all panels: ns, non significant, *p<0.05, **p<0.01, ***p<0.001, ****p<0.0001.
(TIF)

S5 Fig *Hts* and *Gcn5* RNAi validation in *Drosophila*. (A, B) Nephrocyte-specific knockdown of *hts* (A) and *Gcn5* (C) in nephrocytes was performed using using *pros-*GAL4. Dissected garland nephrocytes of the indicated genotypes (see also S1 Table) were stained for Hts (green; A) and Gcn5 (green; C). Nuclei were stained with Hoechst (blue). Scale bars: 10 μm. (B, D) *Hts* (B) and *Gcn5* (D) RNAi knockdown validation was performed with tub-GAL4 and the fat body-specific lpp-GAL4, respectively. Note that the knockdown of Gcn5 with *tub-*GAL4 and *da-*GAL4 was lethal in the embryonic stage and thus could not be used for knockdown validation.
(TIF)

S6 Fig Adducin-α and -γ co-expression in garland nephrocytes. The knockdown of *hts* and simultaneous re-expression of human HA-tagged adducin-α and V5-tagged adducin- γ was performed in garland nephrocytes with *prospero (pros)-GAL4* (see S1 Table for details on transgenic flies). Dissected garland nephrocytes of the indicated genotypes were stained for HA (green) and V5 (red). Nuclei were stained with Hoechst (blue). Scale bar: 10 μm.
(TIF)

S7 Fig Kirre localization in nephrocytes in single and double knockdown of *hts* and *Gcn5*. (A) *Pros-GAL4*-mediated knockdown of *hts* and/or *Gcn5* in garland nephrocytes. Dissected garland nephrocytes of the indicated genotypes were stained for Kirre (red) and Pyd (blue). Scale bar: 10μm. (B) Quantification of nephrocytes showing a continuous Kirre staining using >12 samples/genotype in 3 independent experiments. Statistical analysis was performed with Kruskal Wallis with Dunn’s post-test.
(TIF)

S8 Fig Expression and subcellular localization of adducin- γ and KAT2B in podocytes. (A) Cell lysates from undifferentiated podocytes were analysed by western blotting using anti- adducin-γ. Anti-α-tubulin was used as a loading control. (B) Differentiated podocytes were stained for adducin-γ (green), HA (magenta) and DNA (blue). (C) Undifferentiated podocyte cell lysates were analysed by western blotting using the anti-KAT2B. Anti-α-tubulin was used as a loading control. (D) Differentiated podocytes were stained for KAT2B (green) and HA (red). Scale bars: 20 μm.
(TIF)

S1 Table Fly strains used in this study. (DOCX)
