# Correction: A homozygous *KAT2B* variant modulates the clinical phenotype of *ADD3* deficiency in humans and flies

**DOI:** 10.1371/journal.pgen.1007748

**Published:** 2018-10-26

**Authors:** Sara Gonçalves, Julie Patat, Maria Clara Guida, Noelle Lachaussée, Christelle Arrondel, Martin Helmstädter, Olivia Boyer, Olivier Gribouval, Marie-Claire Gubler, Geraldine Mollet, Marlène Rio, Marina Charbit, Christine Bole-Feysot, Patrick Nitschke, Tobias B. Huber, Patricia G. Wheeler, Devon Haynes, Jane Juusola, Thierry Billette de Villemeur, Caroline Nava, Alexandra Afenjar, Boris Keren, Rolf Bodmer, Corinne Antignac, Matias Simons

The legends for Figs 4 and 5 do not match. The legend that is shown for Fig 4 should be for Fig 5, and the legend that is shown for Fig 5 should be for Fig 4.

**Fig 4:**
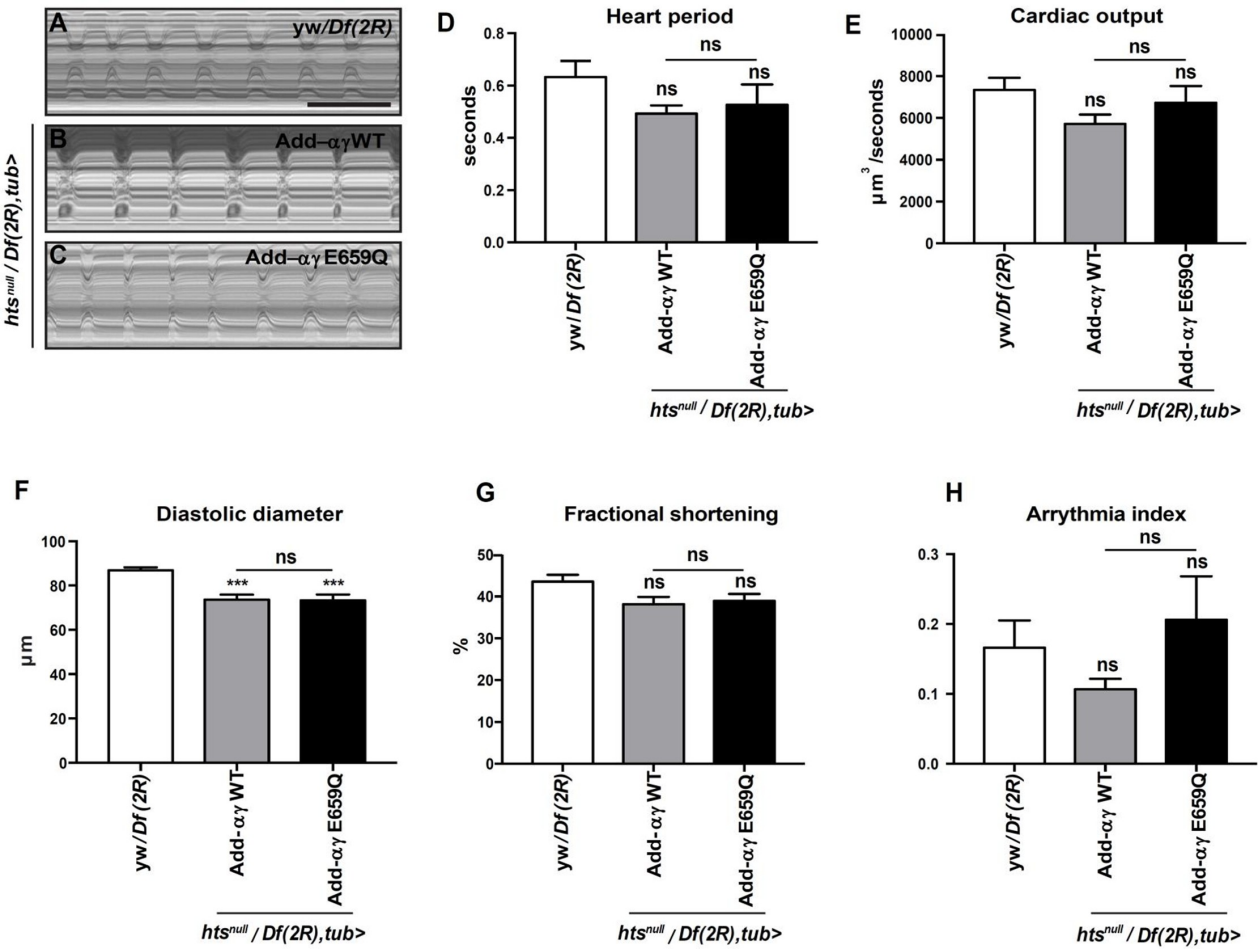
Effect of adducin-αγ E559Q on *Drosophila* heart function. (A-C) M-mode of beating 2-week-old control *(yw/Df(2R)*; A), adducin-αγ WT (B) and adducin-αγ E559Q (C) rescue hearts. Scale bar: 1 second. (D-H) High-speed movies of beating adducin-αγ WT, adducin-αγ E559Q rescue and control hearts were analysed using semi-automated Optical Heartbeat Analysis [46]. For quantification, 8–19 flies were analyzed. Statistical analysis was performed using one-way ANOVA and Tukey’s multiple comparison, except for Arrhythmia index (H; n = 8–19, Mann-Whitney-Wilcoxon). For all panels: ns, non significant, ***p<0.001 (See S1 Table for details on transgenic flies).

**Fig 5:**
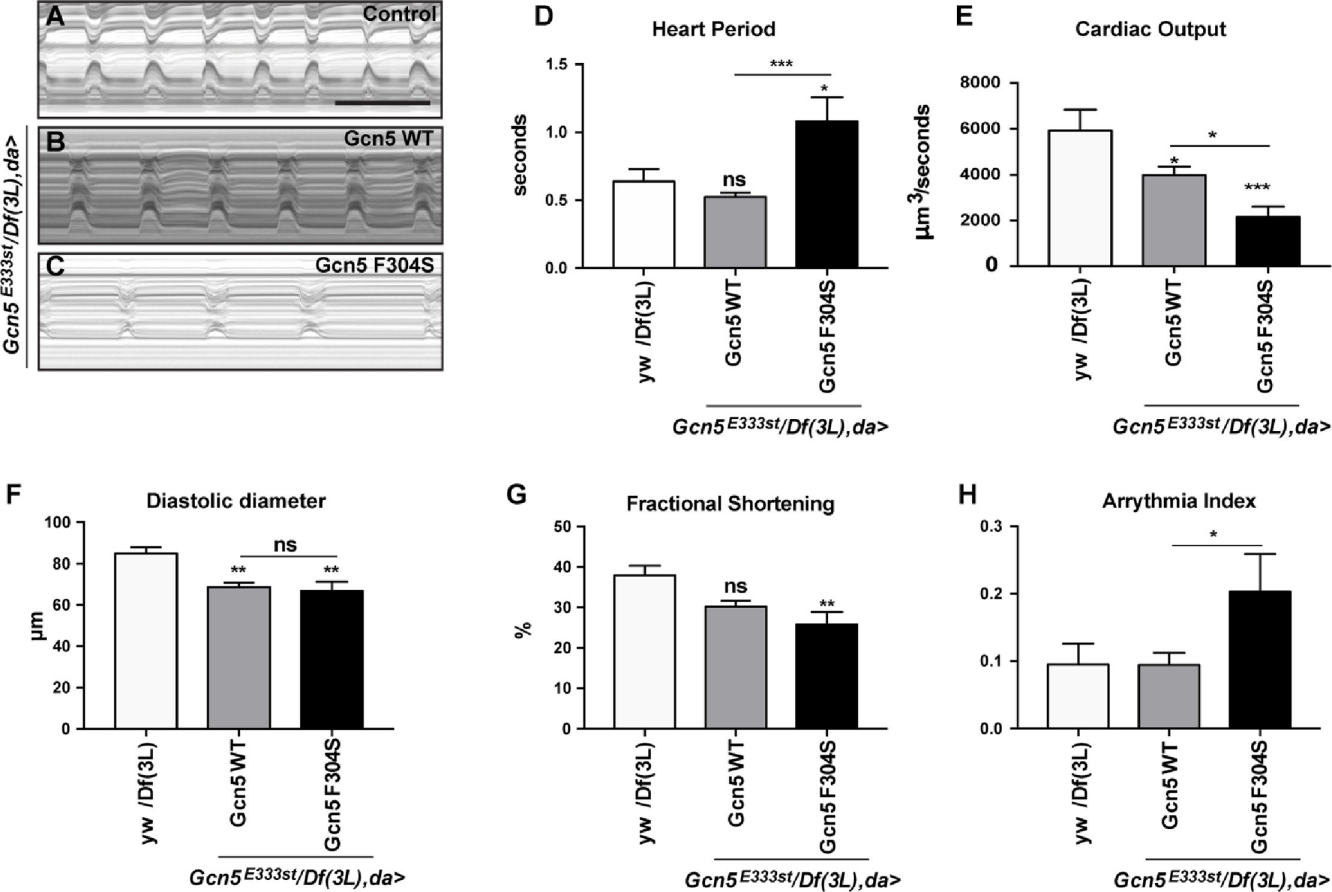
Effect of Gcn5 F304S mutation on *Drosophila* heart function. (A-C) M-mode kymographs of 1 day old beating hearts of control flies *(yw/Df(3L)*; A) and Gcn5^*null*^ flies rescued with Gcn5 WT (B) or Gcn5 F304S (C). Scale bar: 1 second. (D-H) High-speed movies of beating hearts were analysed using semi-automated Optical Heartbeat Analysis [46]. For quantification, 8–19 flies were analyzed. Statistical analysis was performed using one-way ANOVA and Tukey’s multiple comparison for all parameters except arrhythmia index (H), which was analysed using Mann-Whitney-Wilcoxon. For all panels: ns, non significant, *p<0.05 **p<0.01, ***p<0.001, ****p<0.0001 (see S1 Table for details on transgenic flies).

Consequently, the seventh sentence under the subheading “KAT2B F307S but not ADD3 E659Q causes cardiac defects in *Drosophila*” is incorrect. The correct sentence is: Both Gcn5 WT and F304S rescue flies showed a reduction in the normal diastolic diameter compared to control flies (Fig 5F), but only for Gcn5 F304S there was a reduction in contractility, measured as fractional shortening (Fig 5G).

In addition, there are formatting inaccuracies in Table 1. Please see the correct Table 1 here.

**Table 1:** Clinical phenotype of affected individuals.

	Family A			Family B		Family C	Kruer et al^7^
	II-1	II-3	II-6	II-3	II-4	II-1	4 affected sibs (II-1, II-2, II-3, II-4)
**Sex**	F	F	M	NK	F	M	II-1 and II-3: F II-2 and II-5: M
**SRNS**	Yes	Yes	Yes	NK	No	No	No
**Age of onset of proteinuria (yrs)**	7	12	<13	NA	No proteinuria	No proteinuria	NA
**Renal histology**	FSGS	FSGS	FSGS	NA	NA	NA	NA
**Age of ESRD (yrs)**	17	27	13	NA	NA	NA	NA
**Heart disease**	Dilated cardiomyopathy (dx 16 yrs), supra-ventricular arrhythmia (frequent auricular extra-systoles), heart failure	Dilated cardiomyopathy, arrhythmia	Dilated cardiomyopathy (dx 8 yrs), arrhythmia (ventricular hyperexcitation), heart failure	NK	No	No	No
**Neurological features**	Borderline microcephaly, Intellectual disability, MRI: aspects of global demyelination, axonal demyelinating motor-sensory neuropathy	CP: -1SD Intellectual disability	Borderline microcephaly (CP: -2SD), Intellectual disability, MRI: thin corpus callosum	Corpus callosum agenesis	Microcephaly (CP: -3SD), moderate intellectual disability, MRI: partial agenesis of corpus callosum	Microcephaly (CP: -2.4 SD), intellectual disability, intractable seizures, MRI: possible cortical dysplasia	Borderline microcephaly (all sibs), mild to severe intellectual disability (all sibs), spastic plegia (all sibs), thin corpus callosum (II-2), supranuclear gaze palsy (II-2), epilepsy (II-2), convergence-retraction nystagmus and strabismus (II-5), strabismus (II-3)
**Cataract**	Congenital bilateral cataract	Congenital bilateral cataract	Bilateral cataract (6 yrs)	NK	Bilateral cataract	No	NK
**Other features**	Mild facial dysmorphy (wide nasal bridge), arachnodactyly, lax joints, cubitus valgus, scoliosis, short stature	Dysmorphic features (similar to the two brothers)	Facial dysmorphy (wide nasal bridge, slight proptosis) arachnodactyly, *s*hort 4^th^ and 5^th^ metatarsals, conical phalanges, lax joints, cubitus valgus, scoliosis, spread iliac wings, short femural neck, microcytic anemia	NK	mild facial dysmorphy (wide nasal bride, bulbous nasal tip, narrow palpebral fissures) fifth finger mid-phalanx hypoplasia, short 4^th^ and 5^th^ metatarsals, short stature	Facial dysmorphology, short stature	
**Age at last examination *vs*** † **age (yrs)**	† 19	† 28	19	TOP	14	8	16 (II-1) 13 (II-2) 9(II-3) 3(II-5)

Abbreviations are as follows: CP cephalic perimeter; DD, developmental delay; ESRD, end-stage renal disease; F, female; FSGS, focal segmental glomerulosclerosis; yrs, years; M, male; MRI, magnetic resonance imaging NA, not applicable; NK, not known; SRNS, steroidresistant nephrotic syndrome; SD standard deviation; SS, short stature; TOP, termination of pregnancy; yrs, years; †, deceased
