# Supplementary figures and images for: A homozygous KAT2B variant modulates the clinical phenotype of ADD3 deficiency in humans and flies
Source: PLoS Genet. 2018 May 16;14(5):e1007386. doi: 10.1371/journal.pgen.1007386 (PMC5973622; doi:10.1371/journal.pgen.1007386)

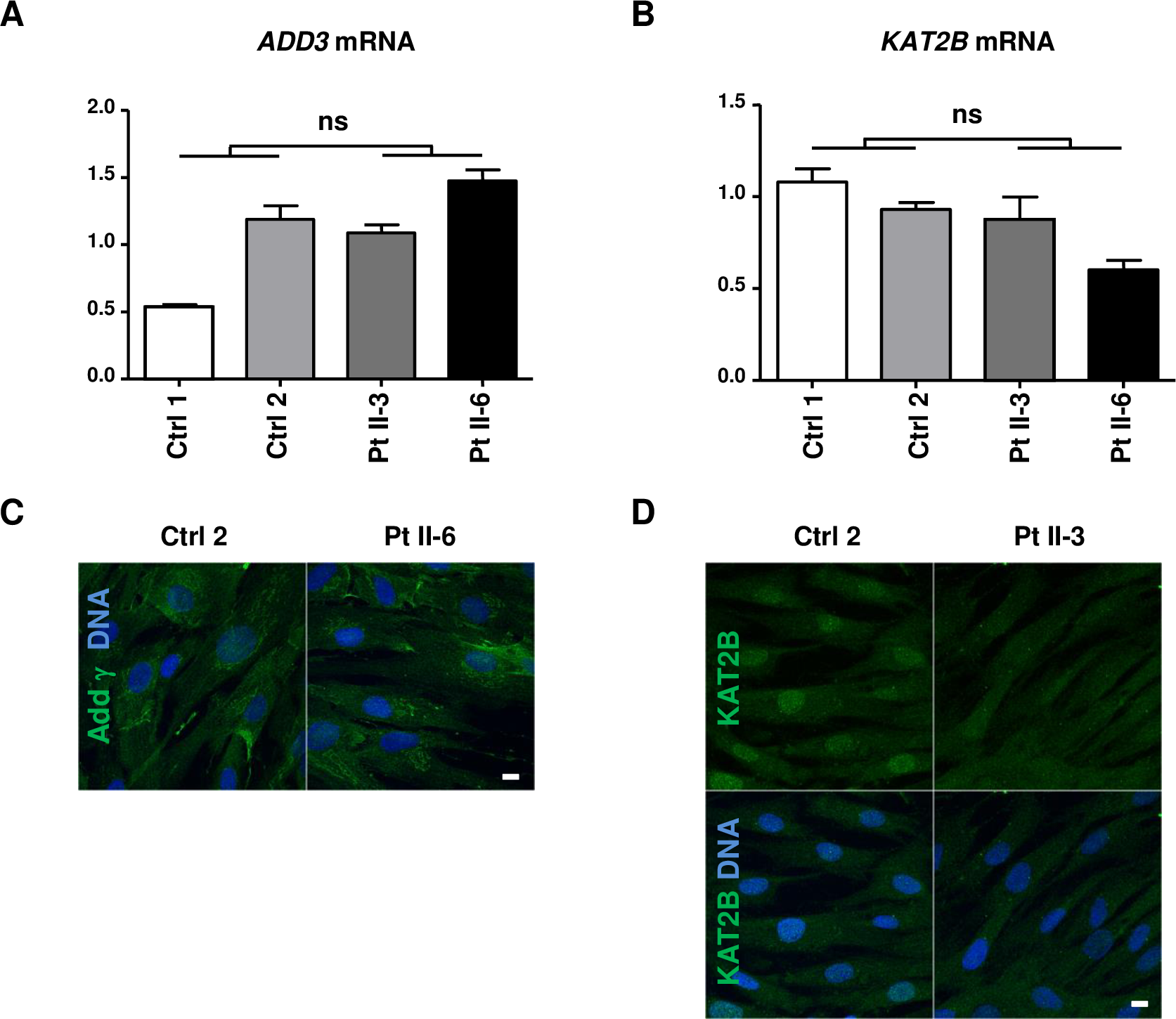

Supplement: S1 Fig — (A, B) ADD3 (A) and KAT2B (B) mRNA levels in patient fibroblasts were assessed by quantitative PCR. Experiments were repeated at least three times and gene expression levels were normalized to the house keeping gene HPRT. Statistical analysis was performed using student’s t-test; ns, non-significant. (C, D) Immunostaining was performed for adducin-γ (green; C) and KAT2B (green; D) in control and patient fibroblasts. Nuclei were stained with Hoechst (blue). Note the loss of nuclear staining for KAT2B in patient fibroblasts. Scale bars: 10 μm. (TIF) [file pgen.1007386.s001.tif]

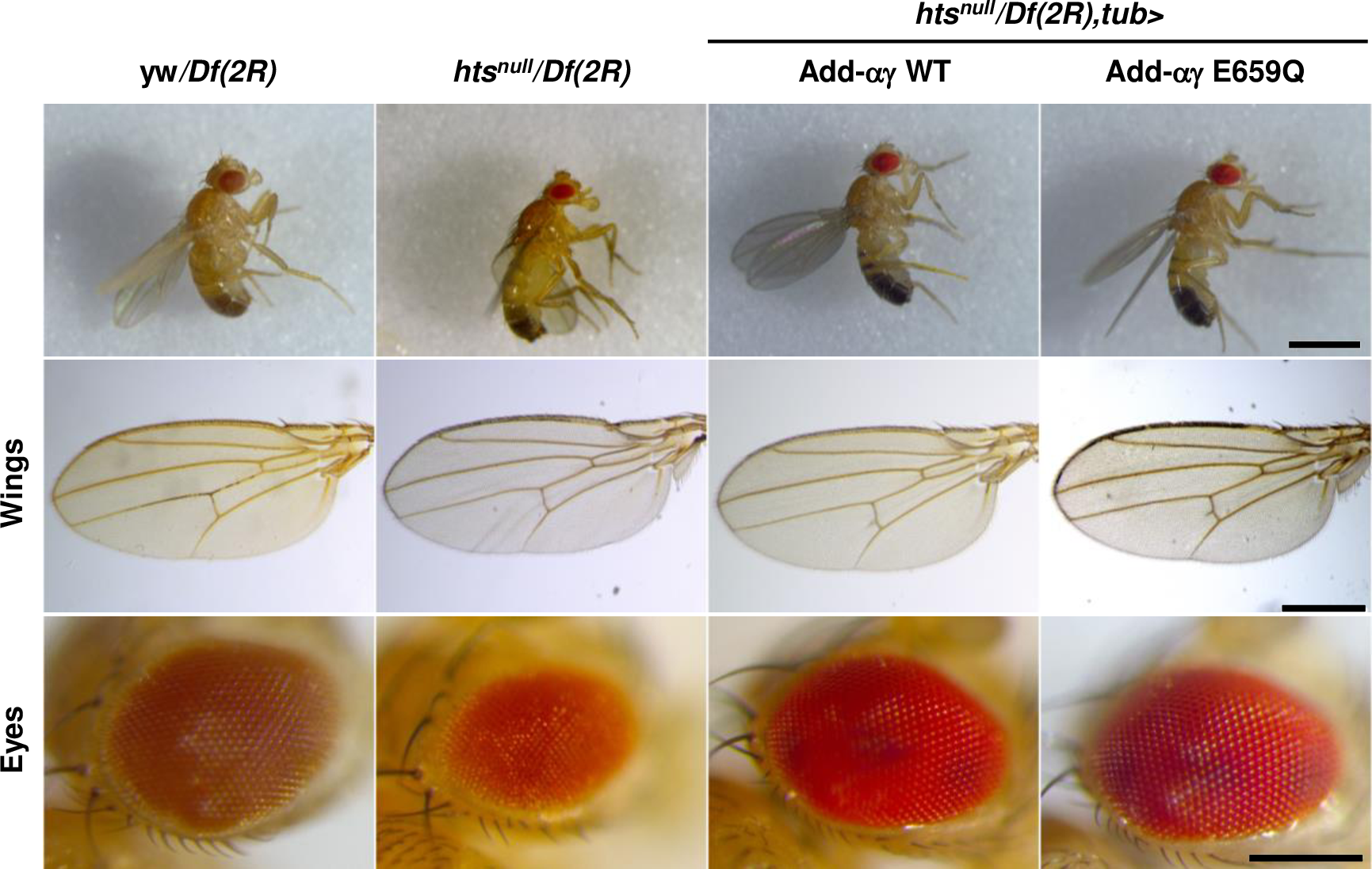

Supplement: S2 Fig — Representative pictures of htsnull and respective adducin-αγ WT and E659Q rescue mutants one day post-eclosion. htsnull flies have rough eye and motor coordination defects and are unable to fly. The ubiquitous co-expression of adducin-α and -γ using tub-GAL4 rescues these defects regardless of the presence of the E659Q mutation. Scale bars: upper panel: 1mm, wings: 500μm, eye: 200μm. (TIF) [file pgen.1007386.s002.tif]

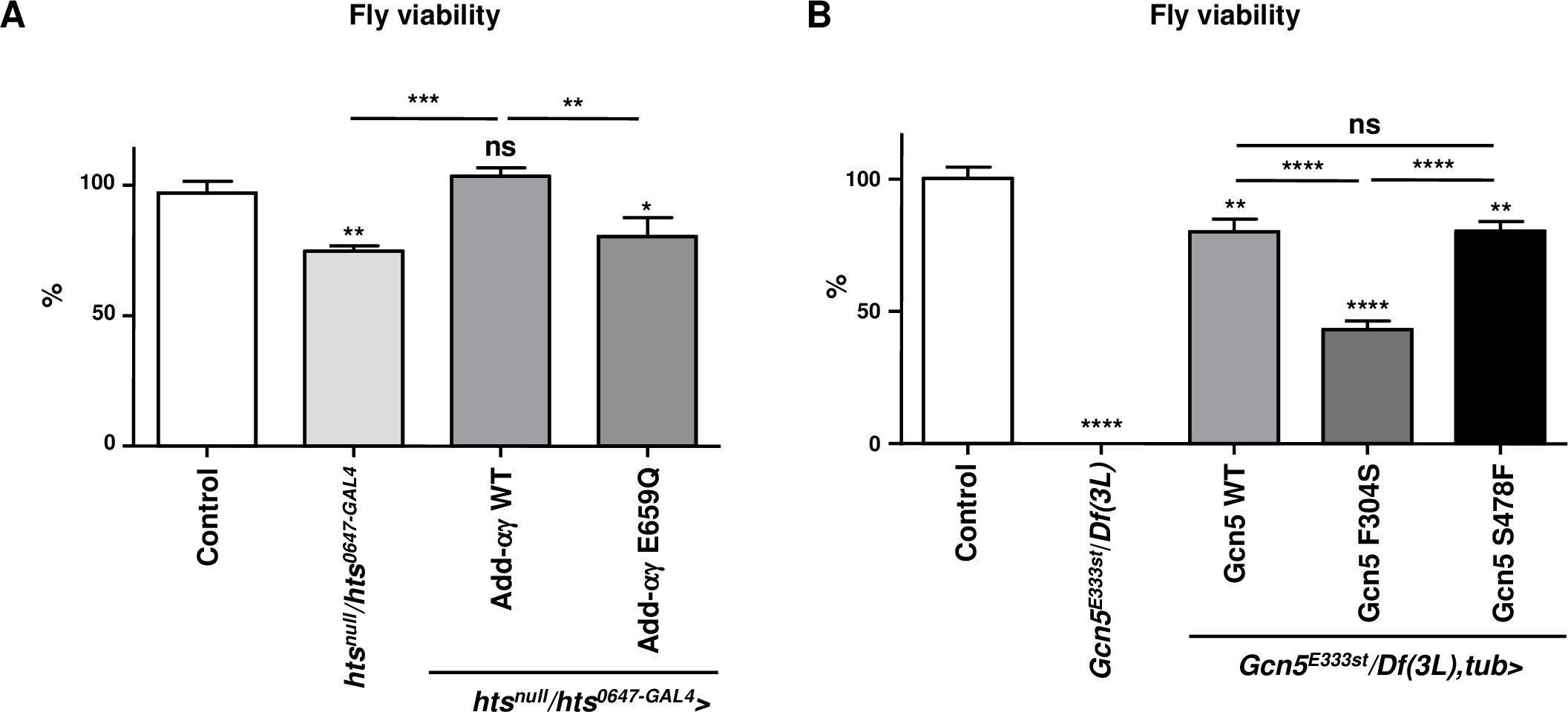

Supplement: S3 Fig — (A) Rescue of hts mutant viability defects with adducin-αγ transgenes driven by a GAL4 insertion in the endogenous hts locus. After 48h of egg laying on standard cornmeal/yeast food, viability was calculated as the percentage of eclosing adults of the indicated genotype and normalized to the control. The control corresponds to the viable F1 trans-heterozygous flies obtained from the cross between Df(2R)BSC26 (harbouring the hts gene) and a non-overlapping deficiency on the same chromosome (Df(2R)247). The insertion of GAL4 in hts locus leads to partial lethality which is completely rescued by adducin-αγ WT but not by E659Q. Quantification is for >100 F1 eclosing flies/genotype/experiment in >5 independent experiments. Statistical analysis was performed using one-way ANOVA with Dunnett’s post-test. (B) Viability for Gcn5null hemizygous flies and respective rescues using tubulin-GAL4 (tub>). Viability was assessed as described in (A). The control corresponds to the viable F1 trans-heterozygous flies obtained from the cross between Df(3L)sex204 (harbouring the Gcn5 gene) and a non-overlapping lethal mutant on the same chromosome (CG31030MI0010). Quantification is for >100 F1 eclosing flies/genotype/experiment in >5 independent experiments. Statistical analysis was performed using one-way ANOVA with Bonferroni post-test. For all panels:ns, non significant, *p<0.05, **p<0.01, ***p<0.001, ****p<0.0001. (TIF) [file pgen.1007386.s003.tif]

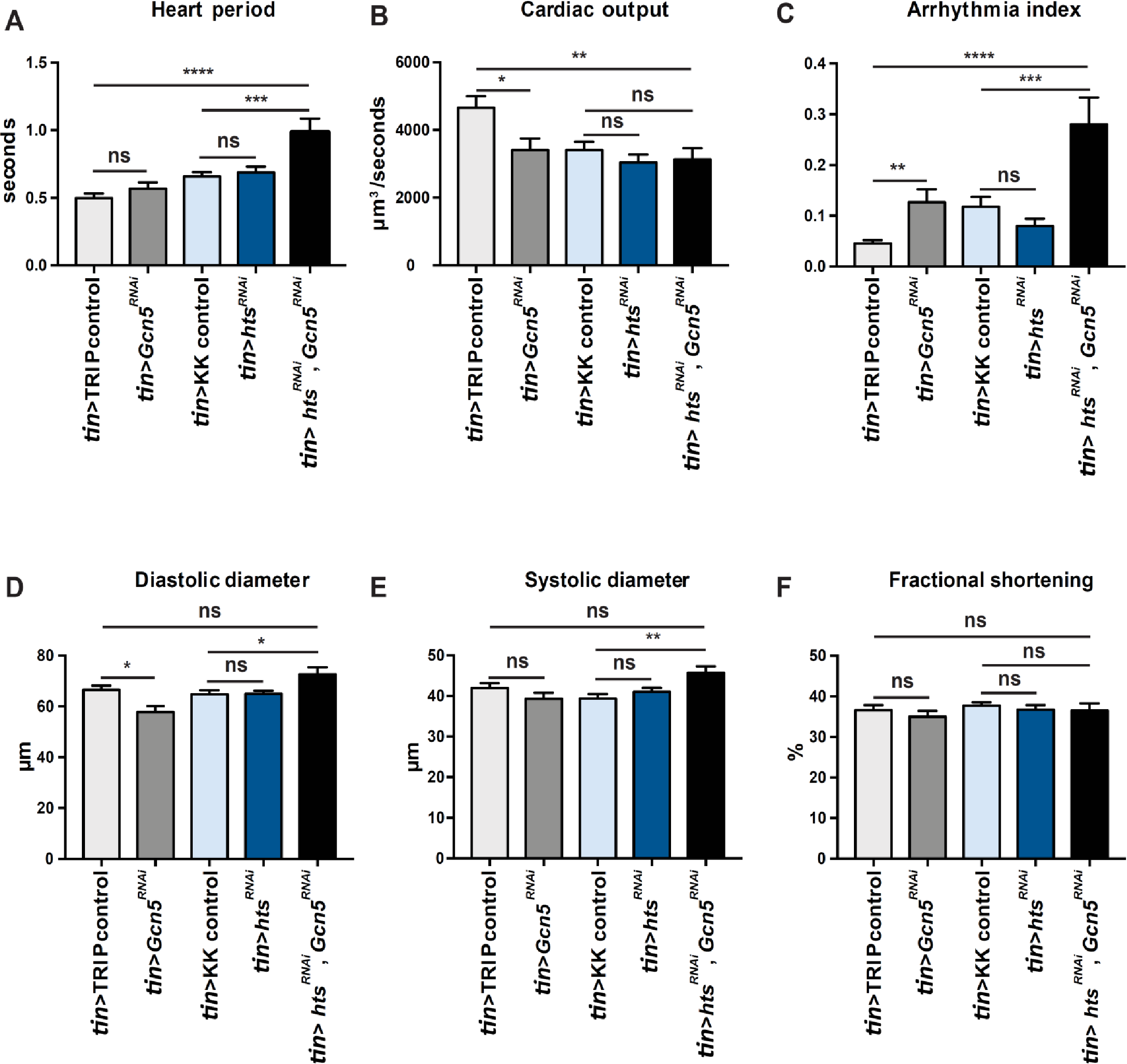

Supplement: S4 Fig — (A-F) Tin-GAL4 driver was used to knockdown hts or/and Gcn5 in cardiomyocytes, and different heart parameters were analyzed in 3 week-old adult flies. Two separate control RNAi lines (TRIP and KK) were used to match Gcn5RNAi (TRIP) and htsRNAi (KK), respectively. For quantification, 19–30 flies were analyzed. Statistical analysis was performed using one-way ANOVA and Tukey’s multiple comparison for all parameters except arrhythmia index, which was analysed using Mann-Whitney-Wilcoxon. For all panels: ns, non significant, *p<0.05, **p<0.01, ***p<0.001, ****p<0.0001. (TIF) [file pgen.1007386.s004.tif]

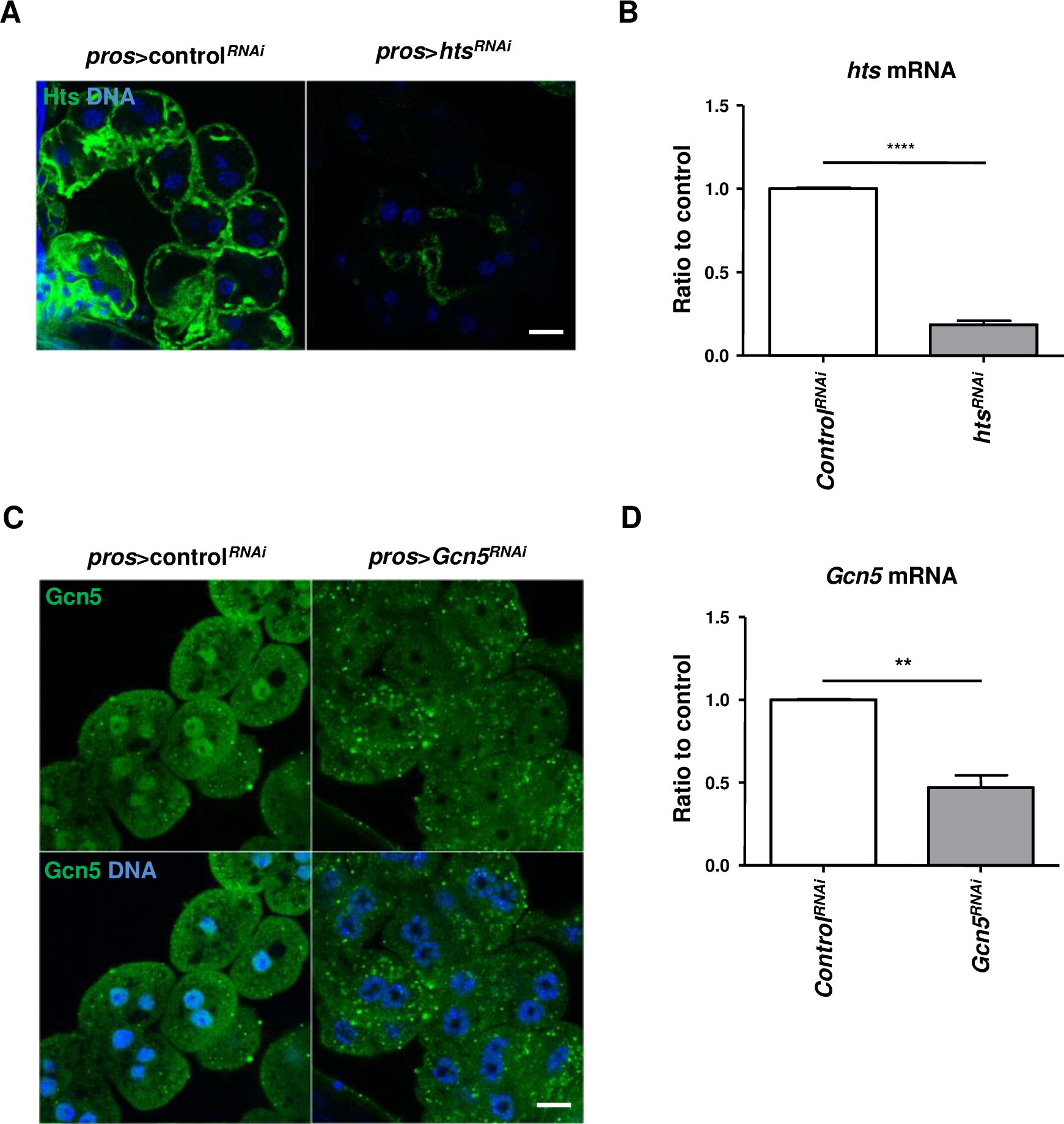

Supplement: S5 Fig — (A, B) Nephrocyte-specific knockdown of hts (A) and Gcn5 (C) in nephrocytes was performed using using pros-GAL4. Dissected garland nephrocytes of the indicated genotypes (see also S1 Table) were stained for Hts (green; A) and Gcn5 (green; C). Nuclei were stained with Hoechst (blue). Scale bars: 10 μm. (B, D) Hts (B) and Gcn5 (D) RNAi knockdown validation was performed with tub-GAL4 and the fat body-specific lpp-GAL4, respectively. Note that the knockdown of Gcn5 with tub-GAL4 and da-GAL4 was lethal in the embryonic stage and thus could not be used for knockdown validation. (TIF) [file pgen.1007386.s005.tif]

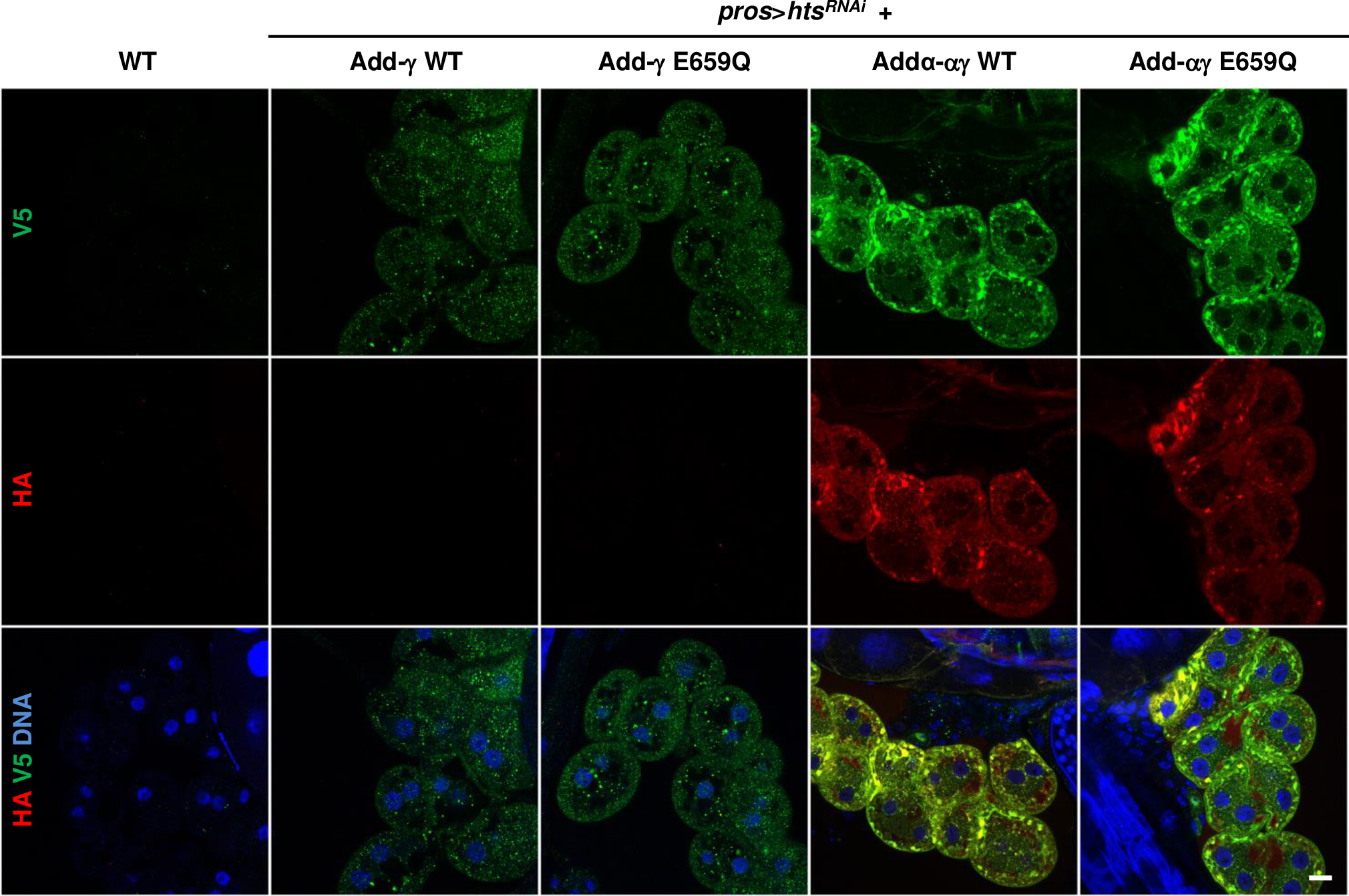

Supplement: S6 Fig — The knockdown of hts and simultaneous re-expression of human HA-tagged adducin-α and V5-tagged adducin- γ was performed in garland nephrocytes with prospero (pros)-GAL4 (see S1 Table for details on transgenic flies). Dissected garland nephrocytes of the indicated genotypes were stained for HA (green) and V5 (red). Nuclei were stained with Hoechst (blue). Scale bar: 10 μm. (TIF) [file pgen.1007386.s006.tif]

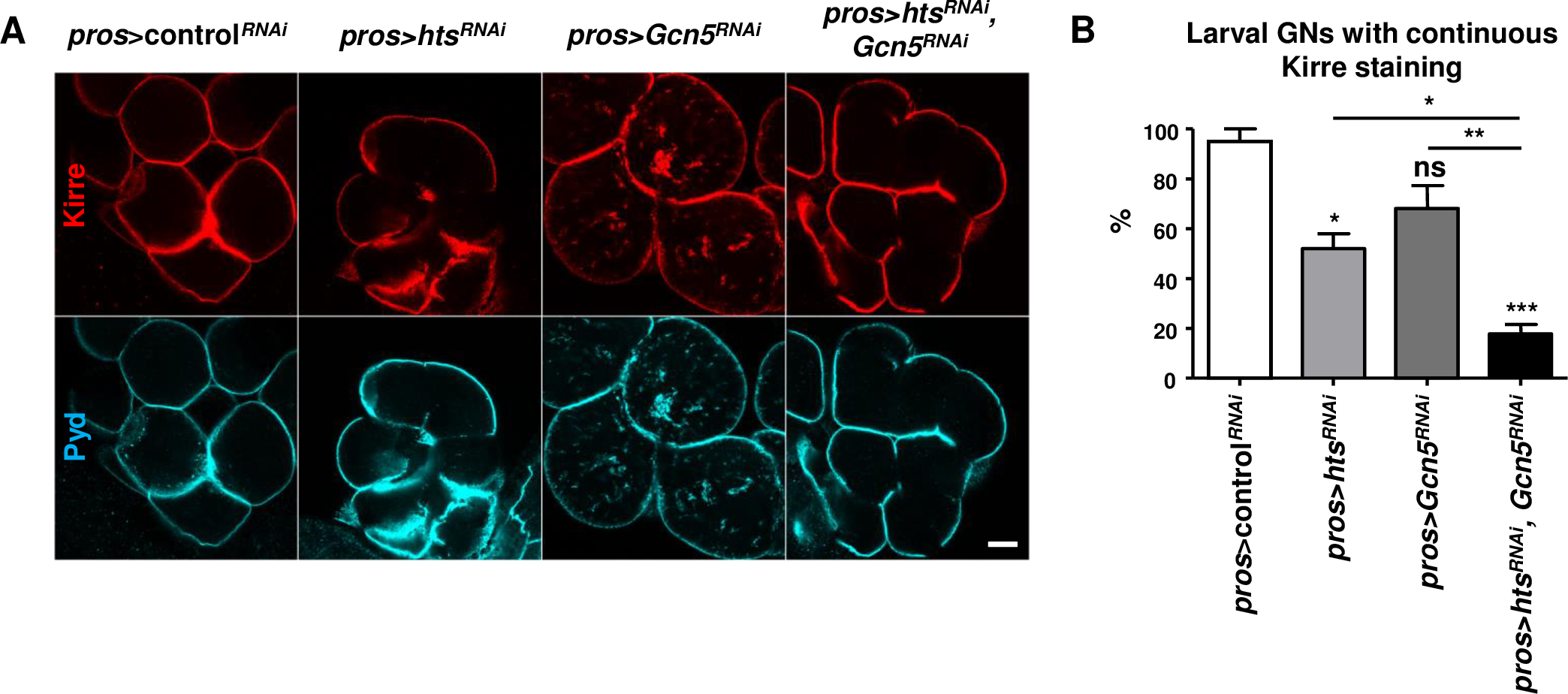

Supplement: S7 Fig — (A) Pros-GAL4-mediated knockdown of hts and/or Gcn5 in garland nephrocytes. Dissected garland nephrocytes of the indicated genotypes were stained for Kirre (red) and Pyd (blue). Scale bar: 10μm. (B) Quantification of nephrocytes showing a continuous Kirre staining using >12 samples/genotype in 3 independent experiments. Statistical analysis was performed with Kruskal Wallis with Dunn’s post-test. (TIF) [file pgen.1007386.s007.tif]

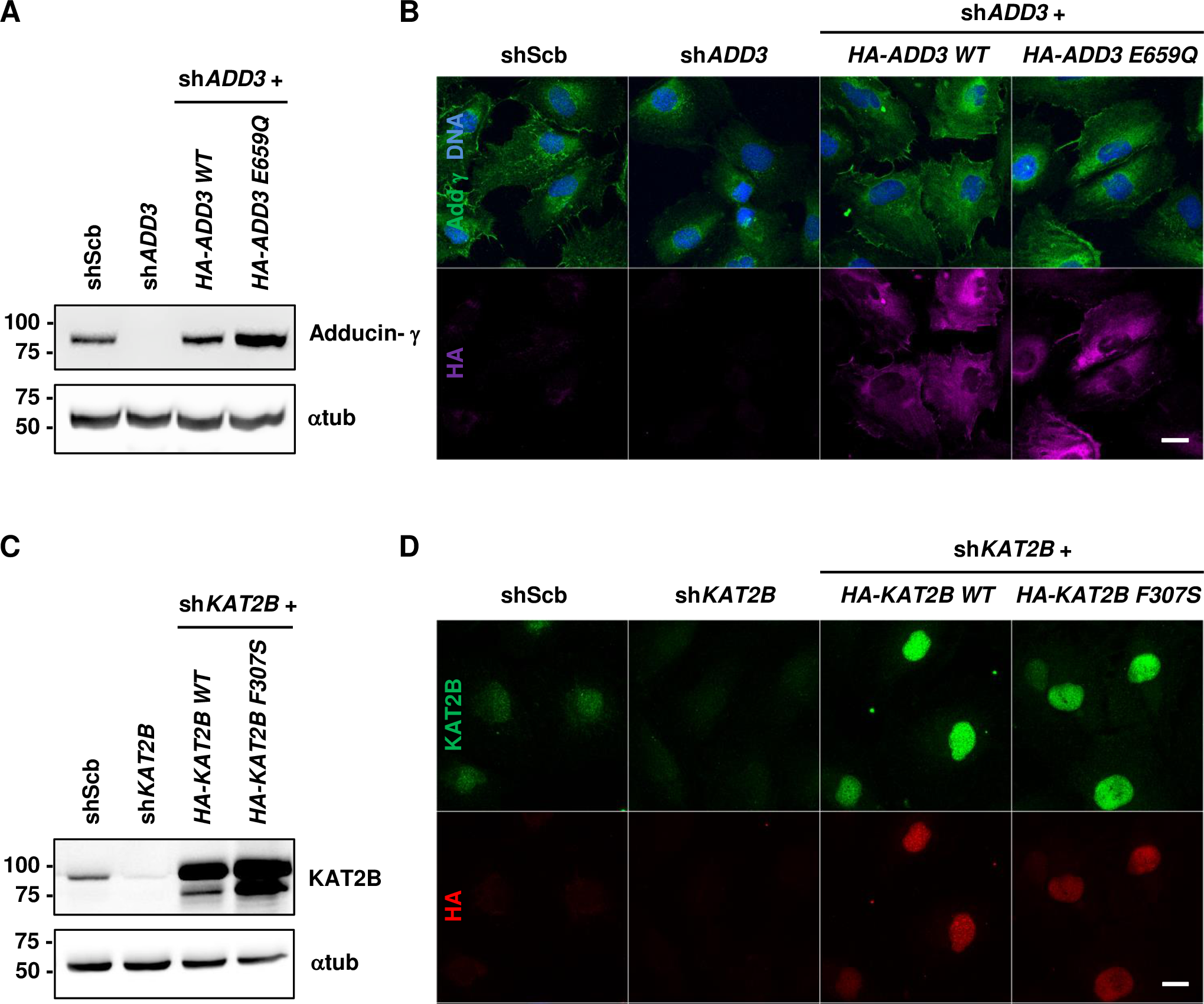

Supplement: S8 Fig — (A) Cell lysates from undifferentiated podocytes were analysed by western blotting using anti- adducin-γ. Anti-α-tubulin was used as a loading control. (B) Differentiated podocytes were stained for adducin-γ (green), HA (magenta) and DNA (blue). (C) Undifferentiated podocyte cell lysates were analysed by western blotting using the anti-KAT2B. Anti-α-tubulin was used as a loading control. (D) Differentiated podocytes were stained for KAT2B (green) and HA (red). Scale bars: 20 μm. (TIF) [file pgen.1007386.s008.tif]
