# Supplementary material for: A homozygous KAT2B variant modulates the clinical phenotype of ADD3 deficiency in humans and flies
Source: PLoS Genet. 2018 May 16;14(5):e1007386. doi: 10.1371/journal.pgen.1007386 (PMC5973622; doi:10.1371/journal.pgen.1007386)
Supplement: S1 Table — (DOCX) [file pgen.1007386.s009.docx]

**S1 Table. Fly strains used in this study**

| **Mutants** | **Origin** | ***GAL4 drivers*** | **Origin** |
| --- | --- | --- | --- |
| *hts^null^* | Takashi Suzuki, University of Tokyo | *tub-*GAL*4* | BL#5138 BDSC |
| *Df(2R)BSC26*  (here referred to as *Df(2R))* | Jan Pielage, University of Kaiserslautern | *da-*GAL4 | Clement Carré, Pierre et Marie Curie University, Paris |
| *Gcn5^E333st^* | Clement Carré, Pierre et Marie Curie University, Paris | *pros-*GAL4 | Barry Denholm, Center for Integrative Physiology, Edinburgh |
| *Df (3L)Sex 204*  (*Df(3L))* | Clement Carré, Pierre et Marie Curie University, Paris | *Dot*-GAL4 | Zhe Han, Children’s Research Institute, Washington |
| *Df(2R)247* | BL#7155 BDSC | *Mhc*-ANF-RFP, *Hand*-GFP; *Dot*-GAL4 | Zhe Han, Children’s Research Institute, Washington |
| *CG31030^MI00107^* | BL#30620 BDSC | *Tin-*GAL4 | Manfred Frasch, Mount Sinai School of Medicine, New York |
| *Hts^0647GAL4^* | BL#63463 BDSC | *lpp-GAL4* | Pierre Leopold, Institut Valrose, Nice |
| **UAS-RNAis** | **Origin** |  |  |
| *UAS-Hts RNAis*  *(Hts^RNAi^)* | KK#103631 VDRC |  |  |
| *UAS-Gcn5 RNAis*  *(Gcn5^RNAi^)* | BL#33981 BDSC |  |  |
| *UAS-TRPP2 RNAi*  *(TRPP2^RNAi^)* | #6941 VDRC |  |  |

Abbreviations: BDSC, Bloomington *Drosophila* Stock Center; VDRC, Vienna *Drosophila* Resource Center

*hts^null^* hemizygous flies were generated by crossing the *hts^null^* flies with *Df(2R)BSC26* flies that carry a deletion overlapping the *hts* locus. Similarly, *Gcn5^null^* hemizygous flies were generated by crossing *Gcn5^E333st^* with *Df(3L)Sex204* flies that carry a deletion of *Gcn5* and two contiguous genes. For rescue experiments, the binary GAL4:UAS system was used to drive the expression of the rescue constructs ubiquitously or in the tissue of interest, on the *hts^null^ or Gcn5^null^* hemizygous or knockdown backgrounds.
